# Glucose-Induced O_2_ Consumption Activates Hypoxia Inducible Factors 1 and 2 in Rat Insulin-Secreting Pancreatic Beta-Cells

**DOI:** 10.1371/journal.pone.0029807

**Published:** 2012-01-03

**Authors:** Mohammed Bensellam, Bertrand Duvillié, Galyna Rybachuk, D. Ross Laybutt, Christophe Magnan, Yves Guiot, Jacques Pouysségur, Jean-Christophe Jonas

**Affiliations:** 1 Pôle d′Endocrinologie, Diabète et Nutrition, Institut de Recherche Expérimentale et Clinique, Université Catholique de Louvain, Brussels, Belgium; 2 Pôle de Morphologie, Institut de Recherche Expérimentale et Clinique, Université Catholique de Louvain, Brussels, Belgium; 3 INSERM U845, Faculté de Médecine, Research Center Growth and Signalling, Université Paris Descartes, Hôpital Necker, Paris, France; 4 Diabetes and Obesity Research Program, Garvan Institute of Medical Research, St. Vincent's Hospital, Sydney, Australia; 5 Unité de Biologie Fonctionnelle et Adaptative, CNRS-Université Paris Diderot-Paris 7, Paris, France; 6 Institute of Developmental Biology and Cancer Research, University of Nice, CNRS UMR 6543, Centre A. Lacassagne, Nice, France; Istituto Dermopatico dell'Immacolata, Italy

## Abstract

**Background:**

Glucose increases the expression of glycolytic enzymes and other hypoxia-response genes in pancreatic beta-cells. Here, we tested whether this effect results from the activation of Hypoxia-Inducible-factors (HIF) 1 and 2 in a hypoxia-dependent manner.

**Methodology/Principal Findings:**

Isolated rat islets and insulin-secreting INS-1E cells were stimulated with nutrients at various pO_2_ values or treated with the HIF activator CoCl_2_. HIF-target gene mRNA levels and HIF subunit protein levels were measured by real-time RT-PCR, Western Blot and immunohistochemistry. The formation of pimonidazole-protein adducts was used as an indicator of hypoxia. In INS-1E and islet beta-cells, glucose concentration-dependently stimulated formation of pimonidazole-protein adducts, HIF1 and HIF2 nuclear expression and HIF-target gene mRNA levels to a lesser extent than CoCl_2_ or a four-fold reduction in pO_2_. Islets also showed signs of HIF activation in diabetic *Lepr^db/db^* but not non-diabetic *Lepr^db/+^* mice. *In vitro*, these glucose effects were reproduced by nutrient secretagogues that bypass glycolysis, and were inhibited by a three-fold increase in pO_2_ or by inhibitors of Ca^2+^ influx and insulin secretion. In INS-1E cells, small interfering RNA-mediated knockdown of *Hif1α* and *Hif2α*, alone or in combination, indicated that the stimulation of glycolytic enzyme mRNA levels depended on both HIF isoforms while the vasodilating peptide adrenomedullin was a HIF2-specific target gene.

**Conclusions/Significance:**

Glucose-induced O_2_ consumption creates an intracellular hypoxia that activates HIF1 and HIF2 in rat beta-cells, and this glucose effect contributes, together with the activation of other transcription factors, to the glucose stimulation of expression of some glycolytic enzymes and other hypoxia response genes.

## Introduction

Hypoxia-Inducible-Factors (HIFs) are basic helix-loop-helix-PAS domain transcription factors composed of a regulated α subunit (HIF1α or HIF2α) and a constitutively expressed HIF1β subunit (Aryl-hydrocarbon-Receptor Nuclear Translocator (ARNT)) [1], [2]. Under normoxic conditions, Prolyl-Hydroxylase-Domain proteins (PHD1-3) hydroxylate HIFα subunits on proline residues in an O_2_-, Fe^2+^- and α-ketoglutarate-dependent manner. This hydroxylation promotes HIFα binding to von Hippel-Lindau protein, followed by their polyubiquitylation and proteasomal degradation. Under hypoxic conditions (O_2_ partial pressure (pO_2_)∼2.3–38 mmHg) or after inhibition of PHDs with CoCl_2_, HIFα subunits are no longer degraded and translocate with ARNT to the nucleus where they activate the transcription of HIF-target genes including glucose transporter 1 (*Glut1*), glycolytic enzymes, monocarboxylate transporter 4 (*Mct4*), the vasodilating peptide adrenomedullin (*Adm*), vascular endothelial growth factors (*Vegfs*), and erythropoietin (*Epo*). This response favours cell survival by triggering a switch from aerobic mitochondrial to anaerobic glycolytic ATP production at the cellular level, an increase in blood flow and capillary growth at the organ level, and an increase in O_2_ transport capacity at the organism level [1]–[3].

The glucose stimulation of insulin secretion (GSIS) by pancreatic beta-cells critically depends on the acceleration of glycolysis and mitochondrial Krebs cycle, with consequent increases in NAD(P)H and ATP production as well as export of Krebs cycle intermediates, including α-ketoglutarate, to the cytosol [4]. Subsequent plasma membrane depolarization and Ca^2+^ influx through voltage-dependent-Ca^2+^-channels trigger insulin granule exocytosis [5]. In addition, glucose stimulates various ATP-consuming processes such as gene transcription, protein synthesis, and Ca^2+^ pumping [6]. In beta-cells, glucose-induced acceleration of ATP production is coupled to an increase in mitochondrial O_2_ consumption [7]–[9]. *In vivo*, the concomitant increase in islet blood flow prevents the fall in intra-islet pO_2_ [10], [11]. In isolated islets maintained *in vitro* or transplanted *in vivo*, however, the glucose stimulation of beta-cell O_2_ consumption leads to a reduction in intra-islet pO_2_ [12]–[14], of which approximately one third depends on the stimulation of Ca^2+^ influx [15]. However, as a drop in pO_2_ and an increase in α-ketoglutarate exert opposite effects on PHD-mediated HIFα hydroxylation [2], it remains unclear whether glucose eventually activates HIFs in beta-cells and, if so, to what extent such activation contributes to the glucose regulation of islet gene expression. In this context, it has recently been shown that glucose activates HIF1 in MIN6 cells and mouse islets only if cultured under slightly hypoxic conditions [16].

Others and we have previously shown that islet expression of hexokinase (*Hk*) 1, lactate dehydrogenase A (*Ldha*), *Mct1* and *4* and Hypoxia up-regulated 1 (*Hyou1*) is increased in hyperglycemic rats [17]–[20]. We more recently reported that the glucose stimulation of cultured rat islets increases their mRNA levels of most glycolytic enzymes (except glucokinase (GK)), of other HIF-target genes like *Adm*, and of genes that are induced by hypoxia independently from HIF activation, like *Hyou1* [21]. We now demonstrate that glucose activates HIF1 and HIF2 in rat beta cells and that both HIF isoforms play distinct roles in the glucose stimulation of expression of glycolytic enzymes and *Adm*. We also provide some evidence that HIFs are activated in islets from diabetic mice, suggesting that hyperglycaemia could induce beta-cell hypoxia *in vivo*.

## Results

### Effects of glucose on HIF-target gene mRNA levels in cultured rat islets and INS-1E cells

To characterize the role of HIF in the glucose stimulation of islet gene expression, we first tested the effect of a 18 h culture in the presence of 2, 5, 10 or 30 mmol/l glucose (G2, G5, G10, or G30) on the mRNA levels of known HIF-target genes and compared it with the effect of HIF activation by CoCl_2_, hypoxia or knockout of *vhlh*, the gene coding the von Hippel-Lindau protein [22].

Due to limited O_2_ diffusion, large islets frequently suffer from central necrosis under normoxic culture conditions [14], [23]. Therefore, islets with central necrosis (usually with a diameter >150 µm) were systematically discarded during preculture, and the islet density per cm^2^ and the medium depth were kept constant between groups. Under these conditions, glucose significantly increased the mRNA levels of many (but not all) HIF-target genes that were up-regulated by more than 2-fold in *vhlh*-knockout islets [22], including *Glut1*, most glycolytic enzymes, *Mct4*, pyruvate dehydrogenase kinase 1 (*Pdk1*), *Adm* and carbonic anhydrase 12 (*Car12*) (Table 1 and Table S1). This effect was larger for genes expressed at low levels under control conditions (*Ldha*, *Adm* and *Car12*) than for the highly expressed genes *Gapdh* and aldolase A (*Aldoa*). Glucose also increased the mRNA levels of *Gapdh*, triose phosphate isomerase 1 (*Tpi1*) and *Adm* (but not *Ldha* that remained below detection limit) in INS-1E cells cultured for 18 h at 70% confluence, indicating that the glucose stimulation of HIF-target gene expression was not restricted to devascularized islets (Table 2). In contrast, glucose failed to affect the islet expression of HIF-target genes that were not or only slightly increased in *vhlh*-KO islets, like hexokinase 1 and 2 (*Hk1* and *Hk2*), and vascular endothelial growth factors (*Vegf*). (Table S1).

**Table 1:** Real-time RT-PCR measurements of glucose-induced changes in HIF subunits and HIF-target gene mRNA levels in cultured rat islets.

*Gene symbol*	C_t_ in G2	Islet *Gene/Tbp* mRNA ratios (relative to G2)			
		G2	G5	G10	G30
*Tbp*	28.0	1±0.10	0.77±0.07	0.67±0.02^a^	0.80±0.04
**HIF subunits**					
*Hif1α*	23.5	1±0.07	0.84±0.03^a^	0.56±0.02^b^	0.40±0.02^b^
*Hif2α*	26.9	1±0.18	0.83±0.10	1.55±0.07	1.71±0.34
*Arnt (Hif1β*	26.7	1±0.02	0.82±0.12	0.59±0.06^a^	0.68±0.11
**Glycolytic enzymes**					
*Aldoa*	21.8	1±0.03	1.25±0.05^b^	0.97±0.07	1.43±0.06^b^
*Tpi1*	25.6	1±0.07	1.12±0.07	1.28±0.15	2.86±0.26^b^
*Gapdh*	22.6	1±0.06	1.21±0.11	1.70±0.14^a^	4.23±0.27^b^
*Eno1*	24.5	1±0.05	1.21±0.26	1.31±0.16	3.47±0.44^b^
*Pkm2*	25.1	1±0.08	0.82±0.03	0.78±0.08	1.27±0.15^c^
*Ldha*	28.6	1±0.15	1.30±0.12	1.59±0.08	4.44±0.30^b^
**Other HIF-target genes**					
*Mct4*	34.0	1±0.12	1.22±0.11	1.54±0.20^a^	1.95±0.07^b^
*Pdk1*	29.0	1±0.04	1.11±0.03	1.03±0.04	3.21±0.05^b^
*Car12*	34.7	1±0.27	1.05±0.46	0.67±0.46	33.2±0.97^b^
*Adm*	31.6	1±0.16	1.28±0.33	0.48±0.19	6.18±0.48^b^

After 1 week preculture in serum-free RPMI medium containing 5 g/l BSA and 10 mmol/l glucose (G10), rat islets were cultured 18 h in the presence of 2, 5, 10 or 30 mmol/l glucose (insulin secretion during culture was (mean ± SEM ng.islet^−1^.h^−1^) 0.06±0.01 in G2, 0.09±0.01 in G5, 0.94±0.22 in G10 and 3.17±0.47 in G30). Glucose-induced changes in the mRNA levels of selected genes were measured by real time RT-PCR. *Tbp* mRNA levels and *Gene/Tbp* mRNA ratios were expressed relative to the level in G2. Data are means ± SEM for 4 experiments. The threshold cycle measured in G2 (real time PCR performed with islet cDNA equivalent to 2 ng total RNA) are shown as rough indicators of gene mRNA levels.

a *p*<0.05,

b *p*<0.01 *vs.* islets cultured in G2;

c *p*<0.01 *vs.* islets cultured in G10 (one-way ANOVA+test of Newman-Keuls).

*Adm*: Adrenomedullin; *Aldoa*: Aldolase A; *Arnt (Hif1b)*: Aryl hydrocarbon receptor nuclear translocator (Hypoxia-inducible factor 1, beta subunit); *Car12*: Carbonic anhydrase 12; *Eno1*: Enolase 1, (alpha); *Gapdh*: Glyceraldehyde-3-phosphate dehydrogenase; *Hif1a*: Hypoxia-inducible factor 1, alpha subunit; *Hif2a*: Hypoxia-Inducible Factor 2, alpha subunit; *Ldha*: Lactate dehydrogenase A; *Mct4 (Slc16a3)*: Monocarboxylate transporter 4 (solute carrier family 16, member 3); *Pdk1*: Pyruvate dehydrogenase kinase, isozyme 1; *Pkm2*: Pyruvate kinase, muscle; *Tbp*: TATA box binding protein; *Tpi1*: Triosephosphate isomerase 1.

**Table 2:** Real-time RT-PCR measurements of glucose-induced changes in HIF subunits and HIF-target gene mRNA levels in INS-1E cells.

*Gene symbol*	C_t_ in G2	*Gene/Tbp* mRNA ratios (relative to G2)			
		G2	G5	G10	G30
*Tbp*	26.4	1±0.06	1.06±0.04	1.02±0.04	1.20±0.05^a^
**HIF subunits**					
Hif1α	25.5	1±0.07	0.74±0.03^b^	0.50±0.04^b^	0.51±0.02^b^
*Hif2α*	24.4	1±0.07	1.23±0.07^b^	1.25±0.04^b^	0.72±0.04^b^
*Arnt (Hif1β)*	24.9	1±0.07	0.96±0.07	1.07±0.13	0.71±0.03
**Glycolytic enzymes**					
*Aldoa*	22.3	1±0.16	1.22±0.08	1.61±0.14^a^	1.47±0.10^a^
*Tpi1*	24.5	1±0.06	0.87±0.03	1.53±0.08^b^	1.71±0.04^b^
*Gapdh*	20.6	1±0.07	1.01±0.04	1.35±0.08^b^	2.06±0.07^b^
*Eno1*	21.5	1±0.12	1.08±0.10	1.52±0.19	2.30±0.14^b^
*Ldha*	ND	ND	ND	ND	ND
**Other HIF-target genes**					
*Pdk1*	29.7	1±0.09	1.01±0.19	1.19±0.11	3.92±0.03^b^
*Adm*	30.7	1±0.19	2.72±0.31^b^	5.24±0.41^b^	4.85±0.40^b^

INS1-E cells were cultured 18 h in the presence of 2, 5, 10 or 30 mmol/l glucose and the mRNA levels of selected genes were measured by real time RT-PCR (insulin secretion during culture was (mean ± SE ng/h, n = 7) 24±2 in G2, 40±7 in G5, 69±15 in G10 and 57±13 in G30). *Tbp* mRNA levels and *Gene/Tbp* mRNA ratios were expressed relative to the level in G2. Data are means ± SE for 3 to 13 experiments. The cycle threshold (C_t_) measured in G2 (real time PCR performed with islet cDNA equivalent to 2 ng total RNA) are shown as rough indicators of gene mRNA levels.

a *P*<0.05,

b *P*<0.01 *vs.* cells cultured in G2 (one-way ANOVA+test of Newman-Keuls).

ND, not detected.

As expected, the mRNA levels of *Gapdh*, *Tpi*, *Ldha*, *Mct4*, *Pdk1* and *Adm* were significantly increased in CoCl_2_-treated rat islets or in islets exposed overnight to hypoxia (pO_2_ ∼38 mmHg) (Table 3), a condition under which all islets developed central necrosis. In contrast, CoCl_2_ and hypoxia did not affect the mRNA levels of genes that are markedly induced by glucose in rat islets but are not HIF-target genes, like thioredoxin interacting protein (*Txnip*) and aldolase B (*Aldob*) (data not shown).

**Table 3:** Effects of CoCl_2_ and hypoxia on the mRNA levels of *Hif1a*, *Hif2a* and several HIF-target genes.

*Gene symbol*	*Gene/Tbp* mRNA ratios			
	(relative to G5)		(relative to G10)	
	*G5*	G5+CoCl_2_	G10	G10-O_2_ 5%
*Hif1α/Tbp*	1±0.08	0.45±0.08^a^	1±0.04	0.97±0.04
*Hif2α/Tbp*	1±0.32	3.48±0.32^a^	1±0.15	1.73±0.15^a^
*Gapdh*/*Tbp*	1±0.04	6.88±0.04^a^	1±0.05	1.33±0.05^a^
*Tpi1/Tbp*	1±0.33	7.00±0.33^a^	1±0.29	4.15±0.29^a^
*Ldha/Tbp*	1±0.42	18.8±0.42^a^	1±0.18	20.6±0.18^a^
*Mct4/Tbp*	1±0.18	8.62±0.18^a^	1±0.19	8.49±0.19^a^
*Pdk1/Tbp*	1±0.15	2.78±0.15^a^	1±0.27	2.27±0.27^b^
*Adm*/*Tbp*	1±0.10	44.2±0.1^a^	1±0.25	34.4±0.25^a^

After preculture, islets were further cultured 18 h in G5 without and with 100 µmol/l CoCl_2_ or in G10 in an atmosphere containing 20 or 5% O_2_. *Gene/Tbp* mRNA ratios were expressed relative to the ratio in G5 or G10. Data are means ± SEM for 3 experiments.

a *p<0.01* and

b *p<0.05 vs.* control islets cultured in G5 or G10 (unpaired student t-test).

### Effects of glucose on the expression of HIF subunits in cultured rat islets and INS-1E cells

To characterize the role of HIF in the glucose stimulation of islet gene expression, we next compared the effects of glucose, CoCl_2_ and hypoxia on the expression of components of the HIF signalling pathway. As shown in Table 1 and Table S1, the mRNA coding the main HIF subunits, HIF-regulating and HIF-interacting proteins were detected in rat islets, some of them being significantly affected by glucose. Most noticeably, glucose (between G2 and G30) decreased *Hif1α* mRNA levels by 60% and *Arnt* mRNA levels by 40% while increasing *Hif2α* mRNA levels 2-fold. Glucose similarly affected *Hif* subunits mRNA levels in INS-1E cells, except that *Hif2α* mRNA levels increased at lower glucose concentrations than in rat islets and tended to decrease between G10 and G30 (Table 2). Interestingly, CoCl_2_ but not hypoxia also decreased *Hif1α* mRNA levels, while both treatments increased *Hif2α* mRNA levels in rat islets (Table 3).

**Figure 1:**
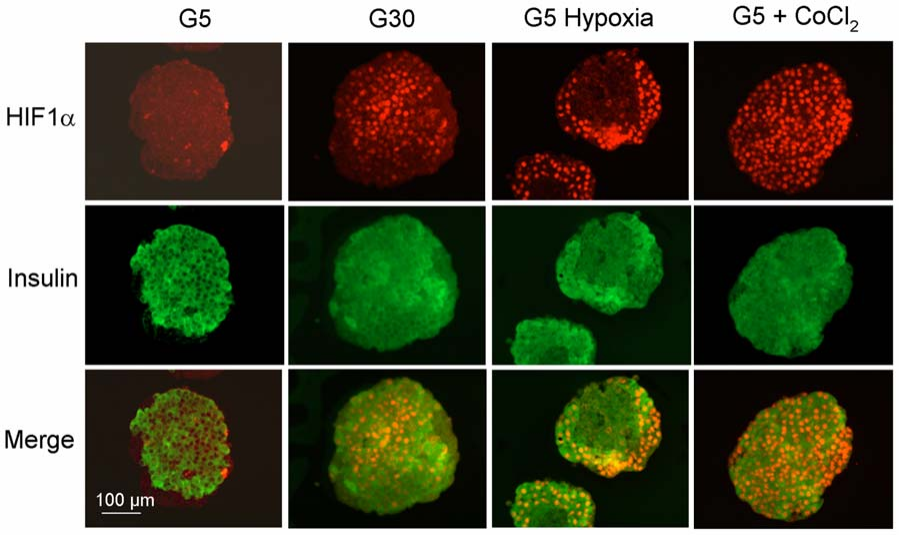
Effects of glucose, hypoxia and CoCl_2_ on HIF1α protein levels in cultured rat islets. After 1 week preculture in G10, rat islets were cultured 18 h in medium containing 5 or 30 mmol/l glucose (G5, G30), G5+200 µmol/l CoCl_2_ or G5 in the presence of 5% O_2_ (pO_2_∼38 mmHg) instead of 20% O_2_ (pO_2_∼151 mmHg) in the incubator (G5 hypoxia). HIF1α and insulin were detected by immunohistochemistry in 5 µm-thick islet sections. Results are representative for 2 to 3 experiments.

Because HIF activation mainly results from the stabilization of its alpha subunits and their nuclear translocation with ARNT, we next tested the effect of glucose, hypoxia and CoCl_2_ on HIF1α and ARNT protein levels in cultured rat islets by immunohistochemistry. After culture in G5, HIF1α was only detected in the nuclei of a few insulin-negative cells while ARNT was detected in the cytosol and nuclei of most islet cells (Figure 1 and Figure S1). After culture in G30, HIF1α staining increased in insulin-positive but not insulin-negative islet cells, while ARNT staining was unaffected. The increase in HIF1α staining was heterogeneous between beta-cells (Figure 1). In comparison, hypoxia and CoCl_2_ markedly increased HIF1α staining in most islet cells outside the central necrotic area. Exposure to CoCl_2_ also tended to increase the intensity of ARNT staining in islet cell nuclei (Figure S1).

**Figure 2:**
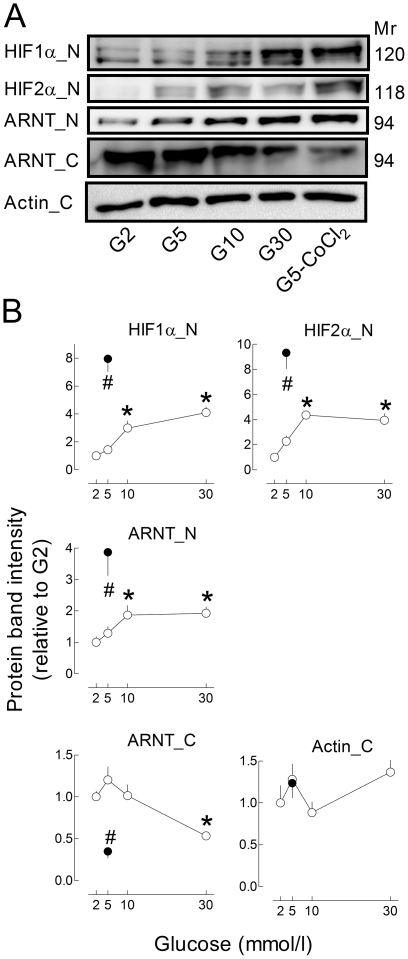
Effects of glucose and CoCl_2_ on HIF1α, HIF2α and ARNT protein levels in INS-1E cells. INS1-E cells (70% confluence) were cultured 18 h in G2, G5, G10, G30 or G5+200 µmol/l CoCl_2_. HIF1α, HIF2α, ARNT (HIF1β) and actin protein levels were measured by Western Blot in nuclear (-N) or cytosolic (-C) extracts. Results are representative blots (A) or means ± SEM of normalized band intensities for 6 to 8 experiments (B). *, *p<0.05* vs. INS1-E cells cultured in G2 (one-way ANOVA+Newman–Keuls test).

Glucose also increased HIF nuclear levels in INS-1E cells (Figure 2). Thus, compared with G2, culture in G30 induced a 4-fold increase in HIF1α and HIF2α nuclear levels and a 2-fold increase in ARNT. Glucose also decreased cytosolic ARNT levels by ∼50%. These glucose effects were, however, of smaller amplitude than those of CoCl_2_ (Figure 2B). These results indicate that, upon glucose stimulation, HIF1α and HIF2α translocate with their dimerization partner ARNT to beta-cell nuclei.

### Effects of Hif1α and Hif2α knockdown on the expression of glycolytic enzymes and Adm in INS-1E cells

**Figure 3:**
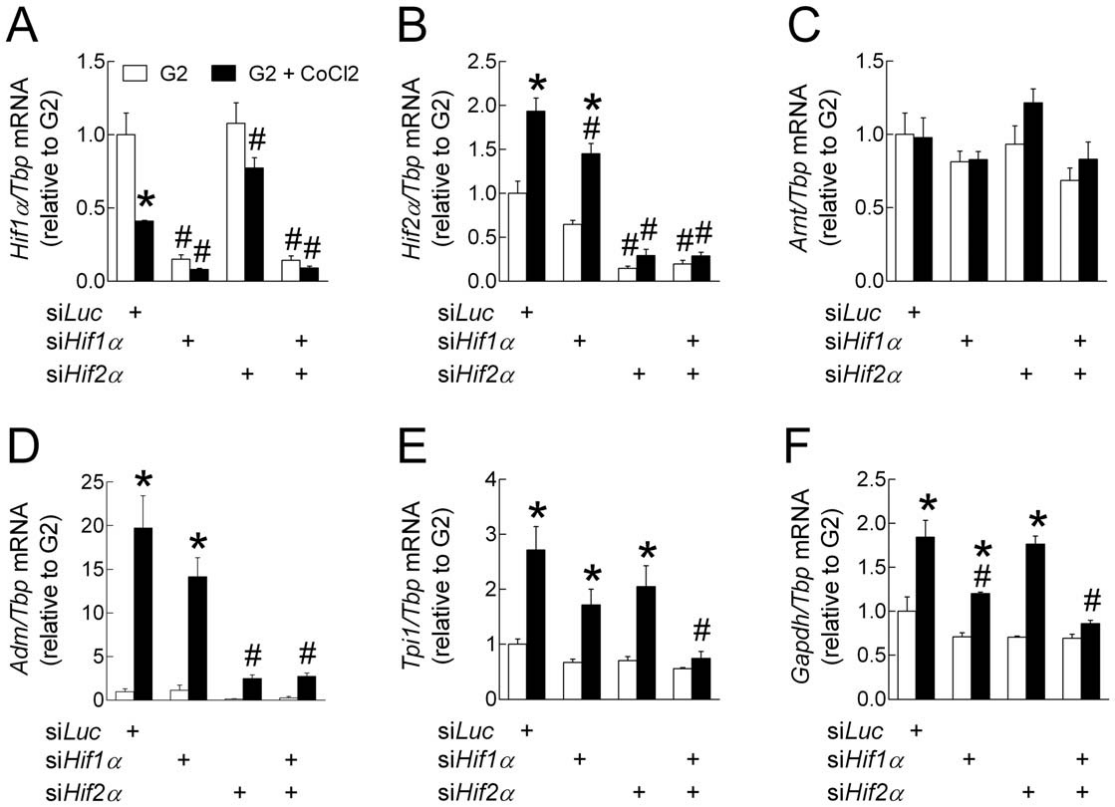
Effects of HIF1α and HIF2α knockdown on the stimulation of HIF-target gene expression by CoCl_2_. INS-1E cells (70% confluence) were transfected for 24 h with siRNA duplexes directed against Luciferase, Hif1α and Hif2α mRNA (siLuc, siHif1α and siHif2α). Then, the transfection medium was replaced with RPMI medium containing 10% foetal calf serum and G2 alone or with 200 µmol/l CoCl_2_. After 18 h culture, the medium was collected for insulin concentration determination and cells were processed for measurement of gene mRNA levels. Gene to Tbp mRNA ratios were expressed relative to the ratio in INS1-E cells treated with siLuc and cultured in G2. Data are means ± SEM for 3 experiments. *, p<0.05 for the effect of CoCl_2_ and #, p<0.05 for the effect of siRNA treatment (two-way ANOVA+test of Bonferroni).

**Figure 4:**
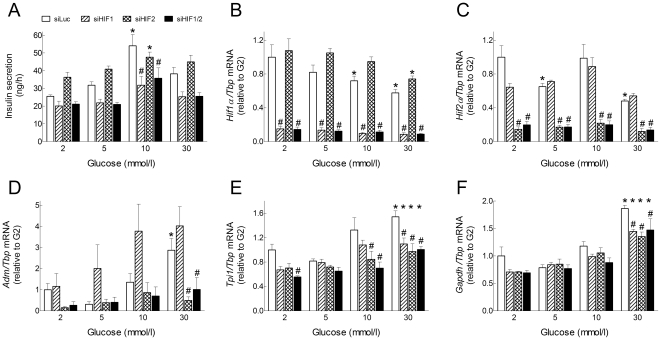
Effects of Hif1α and HIF2α knockdown on the glucose stimulation of HIF-target gene expression. INS-1E cells (70% confluence) were transfected for 24 h with siRNA duplexes directed against Luciferase, *Hif1α* and *Hif2α* mRNA (si*Luc*, si*Hif1α* and si*Hif2α*). Then, the transfection medium was replaced with RPMI medium containing 10% foetal calf serum and increasing glucose concentrations. After 18 h culture, the medium was collected for insulin concentration determination (A) and cells were processed for measurement of gene mRNA levels (B–F). *Gene to Tbp* mRNA ratios were expressed relative to the ratio in INS1-E cells treated with si*Luc* and cultured in G2. Data are means ± SEM for 3 experiments. *, p<0.05 for the effect of glucose *vs.* G2 and *^#^*, *p<0.05* for the effect of siRNA treatment at the same glucose concentration (two-way ANOVA+test of Bonferroni). For *Adm/Tbp* mRNA ratio, the reduction by *siHif2α* in G30 was significant only after removal of *siHif1α* data that were highly variable.

The relationship between HIF1/HIF2 expression and the up-regulation of their target genes was tested in INS-1E cells using small interfering RNAs (siRNAs) against *Hif1α* and *Hif2α*. We first checked the effects of selected siRNAs on the mRNA levels of *Hif* subunits during culture in the presence of G2 and CoCl_2_ (Figure 3A–C) or in the presence of increasing glucose concentrations (Figure 4B–C). As in rat islets, CoCl_2_ significantly decreased *Hif1α* and increased *Hif2α* mRNA levels in INS-1E cells treated with a siRNA against luciferase (siLuc) (Figure 3 B and C). As expected, *Hif1α* and *Hif2α* siRNAs selectively reduced by ∼75% the mRNA levels of the targeted *Hifα* subunit without affecting the other alpha subunit, while their combination markedly reduced both *Hif1α* and *Hif2α* mRNA levels. Interestingly, *Arnt* mRNA levels were not affected by either siRNA.

We next tested the effects of *Hifα* knockdown on CoCl_2_-mediated induction of *Adm*, *Tpi* and *Gapdh* mRNA expression (Figure 3D–F). As expected, CoCl_2_ treatment significantly increased the mRNA levels of the three HIF-target genes in INS-1E cells treated with siLuc. Under these conditions, *Adm* mRNA levels were unaffected by si*Hif1α* but were inhibited by ∼90% by si*Hif2α*. In comparison, the CoCl_2_-mediated stimulation of *Tpi1* and *Gapdh* mRNA expression was unaffected by si*Hif2α* alone, partly reduced by si*Hif1α* alone, and markedly reduced by their combination.

We finally tested the effects of *Hif* knockdown on the glucose stimulation of HIF-target gene expression (Figure 4D–F). Of note, si*Hif1α* significantly reduced GSIS by INS-1E cells while si*Hif2α* tended to increase basal insulin release in G2 and G5 (Figure 4A). Again, si*Hif2α*, but not si*Hif1α*, significantly reduced the glucose induction of *Adm* mRNA expression in INS-1E cells. In comparison, both si*Hif1α* and si*Hif2α*, alone or in combination, only partly reduced the glucose stimulation of *Tpi1* and *Gapdh* mRNA expression. These results indicate that HIF1α and HIF2α are both involved but play distinct roles in CoCl_2_- and glucose-mediated HIF-target gene expression in INS-1E cells.

### Role of the acceleration of mitochondrial metabolism in glucose-induced HIF-target gene expression

**Figure 5:**
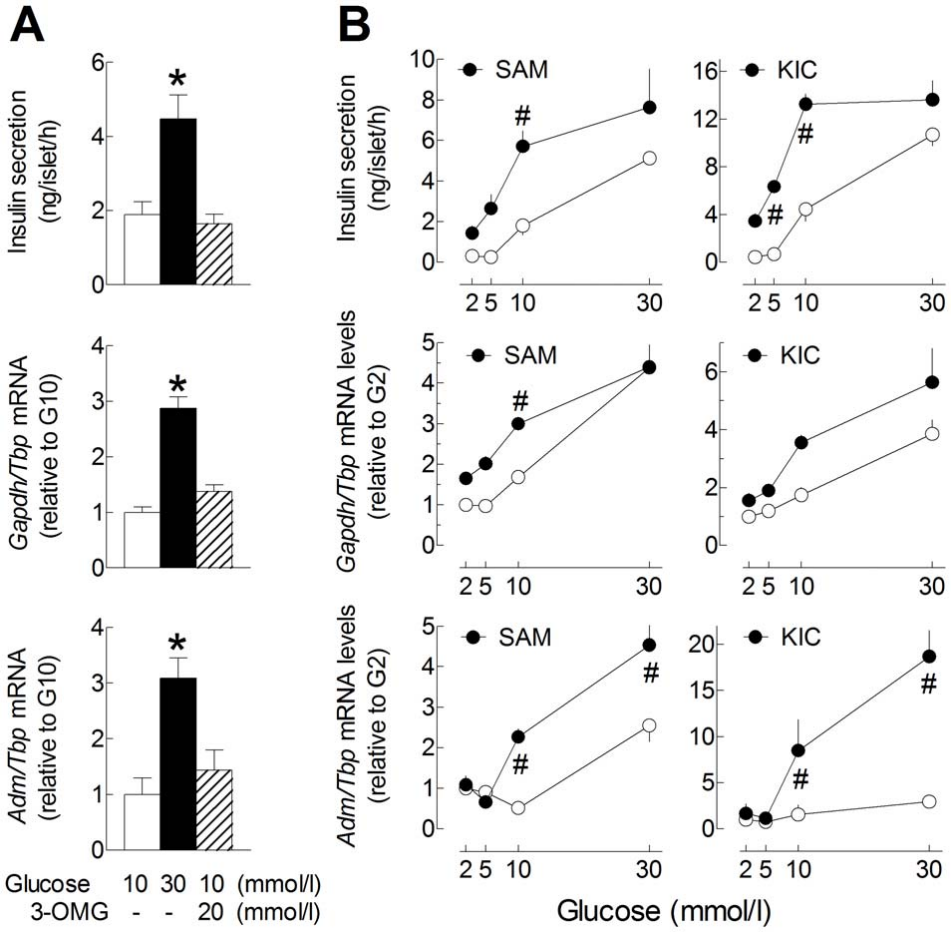
Effects of 3-O-methyl-D-glucopyranose, succinate and α-ketoisocaproate on *Gapdh* and *Adm* expression in cultured rat islets. After preculture, rat islets were further cultured 18 h; A, in the presence of G10 (open columns), G30 (filled columns), or G10+20 mmol/l 3-O-methyl-D-glucopyranose (3-OMG) (hatched columns); B, in the presence of increasing glucose concentrations without (open circle) or with (closed circles) 10 mmol/l succinic acid monomethylester (SAM) or 5 mmol/l α-ketoisocaproate (KIC). *Gene to Tbp* mRNA ratios were expressed relative to the ratio in G10 (A) or G2 (B). Data are means ± SEM for 3 to 6 experiments. The effects of glucose, KIC and SAM on insulin secretion and gene mRNA levels were significant by one-way ANOVA (A) or two-way ANOVA (B)(*P*<0.005). ^#^, *p<0.05* for the effect of SAM or KIC at that particular glucose concentration (two-way ANOVA+test of Bonferroni).

The non-metabolised glucose analogue 3-O-methyl-D-glucopyranose did not reproduce the effect of glucose on insulin secretion, *Gapdh* and *Adm* mRNA levels (Figure 5A). These results indicate that the stimulation of HIF-target gene expression by glucose does not result from a putative osmotic stress but rather depends on its metabolism and activation of downstream events. In agreement, succinic acid monomethyl ester and α-ketoisocaproate, two nutrient secretagogues that bypass glycolysis and directly stimulate mitochondrial metabolism in cultured rat islets, significantly augmented GSIS and the glucose stimulation of *Gapdh* and *Adm* mRNA expression (Figure 5B). Similar results were obtained with a combination of 5 mmol/l leucine and 5 mmol/l glutamine (data not shown). These results are compatible with the hypothesis that the acceleration of mitochondrial metabolism and islet O_2_ consumption with consequent reduction in intra-islet pO_2_ plays a role in the glucose stimulation of HIF-target gene expression.

**Figure 6:**
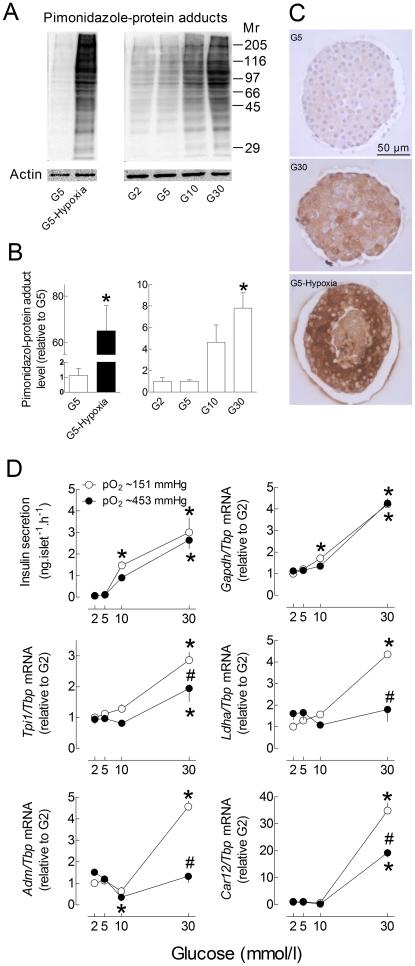
Role of hypoxia in glucose-induced HIF-target gene mRNA expression in cultured rat islets. A–C, after preculture, rat islets were cultured 18 h in G2, G5, G10, G30 or in G5 in the presence of 5% O_2_ (pO_2_∼38 mmHg) instead of 20% O_2_ (pO_2_∼151 mmHg) in the incubator. Pimonidazole was added to the culture medium for the last 2 h and pimonidazole protein-adducts were detected by western blot (A–B) or immunohistochemistry (C). For each lane, the area under the curve (AUC) was calculated and normalized for changes in ACTIN band intensity. Results are representative blots (A) or means ± SEM of normalized AUC for 3–5 experiments (B). *, p<0.05 for the effect of hypoxia (t-test) or glucose *vs.* G2 (one-way ANOVA+Newman–Keuls test). Immunohistochemistry images are representative for 3 experiments (C). D, one week precultured islets were cultured 18 h in G2, G5, G10 or G30 in the presence of 20% O_2_ (pO_2_∼151 mmHg, open circles) or 60% O_2_ (pO_2_∼453 mmHg, close circles). *Gene* to *Tbp* mRNA ratios are expressed relative to the ratio in islets or INS-1E cells cultured in G2 and 20% O_2_. Results are means ± SEM for 3–5 experiments (D). *, p<0.05 for the effect of glucose *vs.* G2 and ^#^, *p<0.05* for the effect of 60% O_2_ at the same glucose concentration (two-way ANOVA+test of Bonferroni).

We therefore used pimonidazole to detect hypoxia in isolated islets cultured in the presence of increasing glucose concentrations. Under hypoxic conditions, reductively-activated pimonidazole forms protein adducts by reacting with cysteine residues independently from the pyridine nucleotide redox state [24]. As shown in figure 6A–C, glucose concentration-dependently increased pimonidazole-protein adducts in cultured islets, but to a much lesser extent than hypoxia. This increase was not restricted to the islet centre and was heterogeneous between islet cells. In comparison, hypoxia triggered central necrosis and strongly increased pimonidazole-protein adducts in surviving cells at the islet periphery.

**Figure 7:**
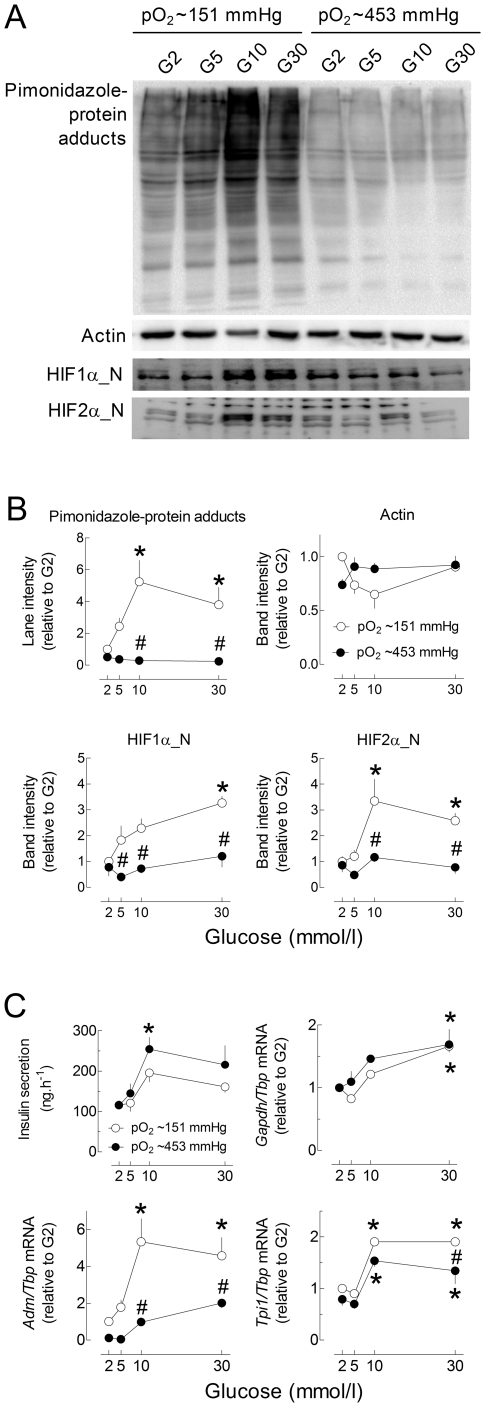
Role of hypoxia in the glucose stimulation of HIF-target gene mRNA levels in INS-1E cells. INS-1E cells (70% of confluence) were cultured 18 h in G2, G5, G10 or G30 in the presence of 20% O_2_ (pO_2_∼151 mmHg, open circles) or 60% O_2_ (pO_2_∼453 mmHg, close circles). Pimonidazole was added to the culture medium for the last 2 h and pimonidazole protein-adducts were detected by western blot (A). For each lane, the area under the curve (AUC) was calculated and normalized for changes in ACTIN band intensity. HIF1α and HIF2α protein levels were measured in nuclear extracts and normalized for changes in ACTIN (A–B). Gene to Tbp mRNA ratios are expressed relative to the ratio in INS-1E cells cultured in G2 and 20% O_2_ (C). Results are representative blots (A) or means ± SEM of normalized AUC for 3–5 experiments (B–C). *, p<0.05 for the effect of glucose *vs.* G2 and #, p<0.05 for the effect of 60% O_2_ at the same glucose concentration (two-way ANOVA+test of Bonferroni).

As the glucose-stimulation of HIF-target gene expression likely results from hypoxia-mediated HIF activation, we next tested the effect of a 3-fold increase in pO_2_ on the glucose stimulation of HIF-target gene expression. As shown in figure 6D, glucose stimulated insulin secretion and *Gapdh* mRNA expression to a similar extent under control and hyperoxic conditions. In contrast, glucose increased *Tpi1*, *Ldha*, *Adm* and *Car12* mRNA levels to a significantly lesser extent under hyperoxic conditions, supporting the role of the increase in islet O_2_ consumption in these glucose effects. Of note, the glucose stimulation of pimonidazole-adduct formation was also suppressed by culture in the presence of 90% O_2_ (Figure S2). Also in INS-1E cells, culture under hyperoxic conditions markedly reduced the glucose stimulation of pimonidazole-protein adduct formation, HIF1α and HIF2α nuclear accumulation, and *Adm* and *Tpi1* mRNA expression (Figure 7). It did not, however, significantly affect GSIS and the stimulation of *Gapdh* mRNA expression. These results indicate that, depending on the HIF-target gene studied, the glucose stimulation of mRNA expression is independent (*Gapdh*) or partly results from hypoxia (*Tpi*, *Adm*), not only in isolated islets, but also in INS-1E cells.

### Role of Ca^2+^ influx in the glucose stimulation of HIF-target gene mRNA expression

**Figure 8:**
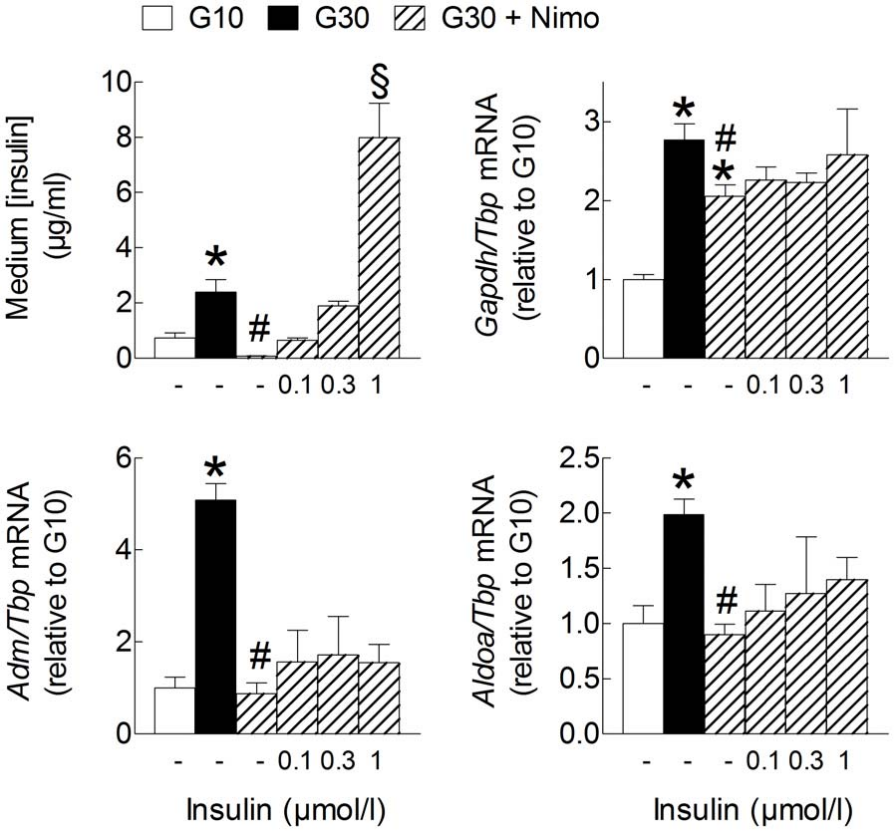
Role of Ca^2+^ influx and insulin secretion in glucose-induced HIF-target gene expression in rat islets. After preculture, islets were cultured 18 h in G10, G30, G30 +1 µmol/l nimodipine (Nimo) or the latter condition plus increasing concentrations of exogenous human insulin (Actrapid), as indicated. The absolute rate of insulin secretion (ng.islet^−1^.h^−1^) in the first three conditions were: 1.02±0.25 in G10; 2.78±0.93 in G30 and 0.09±0.02 in G30+Nimo (n = 3). *Gene to Tbp* mRNA levels were expressed relative to the level in G10. Data are means ± SEM for 3 to 4 experiments. *, *p*<0.05 for the effect of glucose, ^#^, *p*<0.05 for the effect of nimodipine, ^§^, *p*<0.05 for the effect of insulin (one-way ANOVA+test of Newman-Keuls).

It has previously been shown that approximately one third of the glucose stimulation of islet O_2_ consumption is Ca^2+^-dependent [15]. The L-type Ca^2+^ channel blocker nimodipine, which almost fully inhibited insulin secretion during culture in G30, only slightly reduced the mRNA levels of *Gapdh* but markedly reduced *Aldoa* and *Adm* mRNA levels in G30 (Figure 8). Interestingly, the latter inhibition was not prevented by addition of exogenous insulin to the medium, indicating that Ca^2+^ influx contributes to the stimulation of HIF-target gene expression independently from changes in insulin concentration. In contrast, nimodipine exerted opposite effects on *Txnip* and *Aldob* mRNA levels (data not shown). Similar results were obtained with diazoxide, a K_ATP_ channel opener that inhibits glucose-induced Ca^2+^ influx and insulin secretion [25] (data not shown). Also in INS-1E cells, nimodipine significantly reduced the glucose stimulation of *Adm* and *Aldoa* mRNA expression without affecting that of *Gapdh* (Figure S3).

### Hypoxia-mediated HIF activation in islets from diabetic mice?

**Figure 9:**
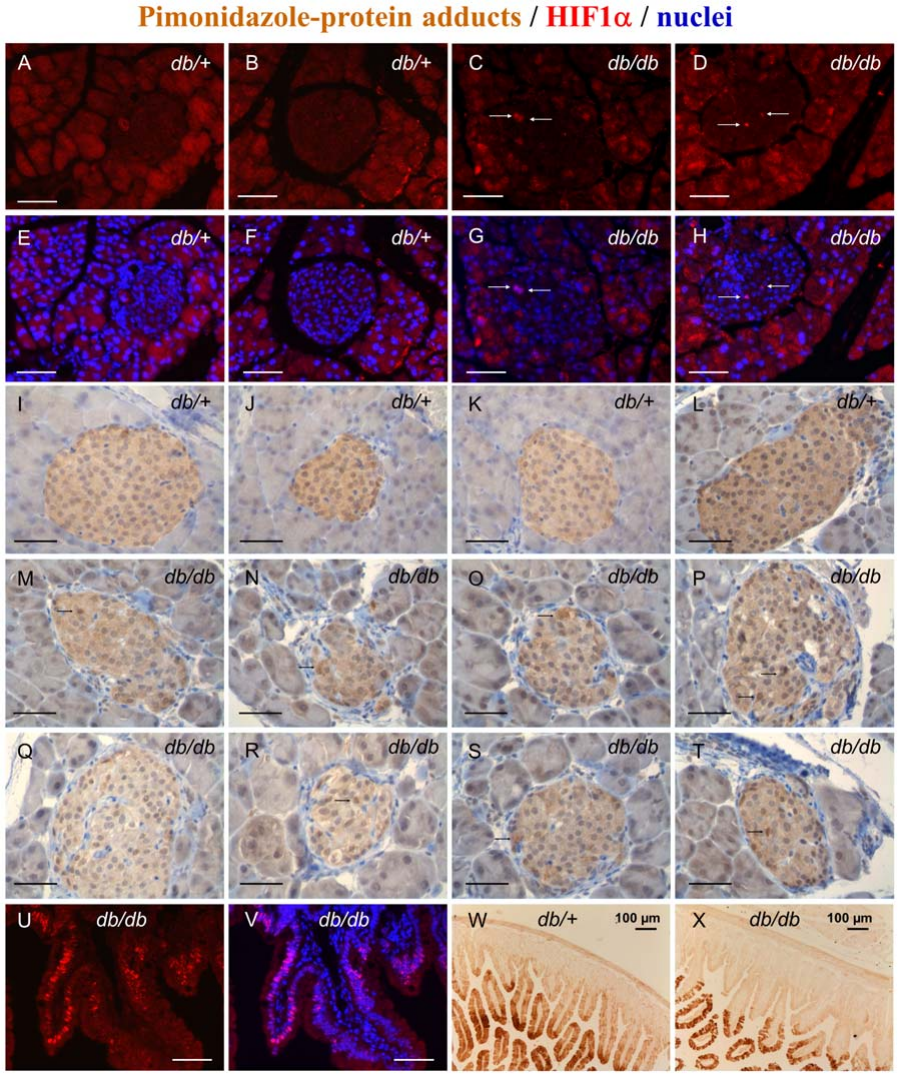
HIF1α protein expression and pimonidazole-protein adduct formation in the pancreas and intestine of diabetic mice. Diabetic *Lepr^db/db^* mice (*db/db*) and non-diabetic *Lepr^db/+^* mice (*db/+*) were killed and their pancreas, spleen and duodenal loop were dissected and fixed in paraformaldehyde solution (4%) overnight at 4°C. A–D, HIF1α expression in 10 week-old male *db/db* mice (n = 2; blood glucose = 25.4 and 24.1 mmol/l; body weight = 45 and 44 g) and non-diabetic *db/+* mice (n = 2; blood glucose = 6.3 and 7.9 mmol/l, n = 2; body weight = 23 and 26 g). E–H, corresponding images with merged HIF1α (red) and Hoechst 33342 (blue) nuclear staining. White arrows, HIF1α-positive islet cell nuclei (purple on merged images). Bar scale = 50 µm. Results are representative for two animals of each kind. I–T, pimonidazole protein-adducts in 26 week-old male and female *db/db* mice (M–T) (n = 3; blood glucose = 25.3±0.9 mmol/l; body weight = 54±3 g) and non-diabetic *db/+* mice (I–L) (n = 3; blood glucose = 6.6±0.3 mmol/l; body weight = 28±0.9 g) injected with pimonidazole (60 mg/kg) 24 h before death (brown 3,3′-diaminobenzidine precipitate). Black arrows, cells with higher pimonidazole-protein adduct intensity. Bar scale = 50 µm. Results are representative for two animals of each kind. U–X, immunodetection of HIF1α and pimonidazole-protein adducts in the duodenal mucosa of *db/+* or *db/db* mice.

To test whether *in vivo* hyperglycaemia also induces hypoxia and activates HIF in pancreatic islets, we first measured HIF1α protein levels in islets from diabetic *Lepr^db/db^* and non-diabetic *Lepr^db/+^* mice. Interestingly, a few HIF1α-positive nuclei were detected in some islets from diabetic mice, whereas none were observed in sections from non-diabetic mice (Figure 9A–H). That the lack of HIF1α staining did not result from a problem in tissue fixation/processing was confirmed by the observation, on the same section, of a large number of HIF1α-positive epithelial nuclei in the villi of the intestinal mucosa (Figure 9U–V). For technical reasons, we could not determine whether the few HIF1α-positive islet cells detected in *Lepr^db/db^* mice are beta-cells or not.

We next measured pimonidazole-protein adducts in the same model of diabetes. Interestingly, islets from both diabetic and non-diabetic mice were more heavily stained than the surrounding exocrine acini (Figure 9I–T), suggesting that they experience low intensity hypoxia irrespective of the glucose tolerance status. This staining seemed, however, less intense than that observed in the villi of the duodenal mucosa (Figure 9W–X). Although the intensity of pimonidazole staining looked similar or even slightly lower in islets from diabetic *vs.* non-diabetic mice, there were clear differences regarding its heterogeneity between cells. Thus, pimonidazole staining was almost uniform throughout the islets of non-diabetic mice (Figure 9I–L), whereas it was heterogeneous in islets from diabetic mice, with a few cells displaying a higher intensity (Figure 9M–T). Unfortunately, we could not determine whether these islet cells with higher pimonidazole staining are beta-cells or not.

We finally measured the islet mRNA levels of *Hifα* subunits and of several HIF-target genes in *Lepr^db/db^* and *Lepr^db/+^* mice. As shown in Table 4, the mRNA levels of Hif1α were not different in islets from *Lepr^db^* ^/*db*^ and *Lepr^db/+^* mice, but those of *Hif2α* and of all HIF-target genes tested (except for *Ldha* tested in a previous study [26]), were significantly up-regulated in the islets of *Lepr^db^* ^/*db*^ mice, further supporting the hypothesis that HIF1 and HIF2 are activated in islets from diabetic mice.

**Table 4:** Changes in the mRNA levels of *Hif1a*, *Hif2a* and several HIF-target genes in islets isolated from diabetic mice.

*Gene symbol*	*Gene/Cyclophilin* mRNA ratios	
	(relative to *Lepr^db^* ^/*+*^)	
	*Lepr^db^* ^/*+*^	*Lepr^db/db^*
*Hif1α*	1±0.14	1.26±0.10
*Hif2α*	1±0.15	3.31±0.53^a^
*Tpi1*	1±0.06	2.19±0.20^a^
*Gapdh*	1±0.05	2.69±0.47^a^
Eno1	1±0.13	3.91±0.45^a^
*Adm*	1±0.12	3.24±0.58^a^
*Hyou1*	1±0.06	3.52±0.47^a^

*Gene/Cyclophilin* mRNA ratio were measured by real-time PCR using islet cDNA from 10-week-old diabetic *Lepr^db^* ^/*db*^ mice and their non-diabetic *Lepr^db^* ^/+^ littermates (fed whole blood glucose levels ∼20 mmol/l and ∼6 mmol/l respectively)(primer sequences are shown in Table S3). The metabolic characteristics of the mice and the mRNA levels of other β-cell genes (including *Hk1* and *Ldha*) in these series of experiments have been reported earlier [26]. Data are means ± SE for 5–9 mice and are expressed relative to the mRNA ratio in control *Lepr^db^* ^/+^ mice.

a *p*<0.01 *vs.* control by Student t-test.

## Discussion

This study demonstrates that, even in small islets and cell monolayer, glucose and other nutrient secretagogues that bypass glycolysis activate HIF1 and HIF2 in rat beta-cells following the induction of a state of moderate hypoxia. This effect, which was not simply due to limited O_2_ diffusion in culture but also depended on the glucose stimulation of O_2_ consumption in beta-cells, contributed to the stimulation of expression of glycolytic enzymes and other hypoxia-response genes. In a recent study very similar to ours but carried out in MIN6 cells and mouse islets, glucose stimulation rapidly (within one hour) triggered beta-cell hypoxia only if the pO_2_ was reduced from 20 to 10% [16]. Despite this difference, both studies converge in showing that in vitro glucose stimulation of beta-cell O_2_ consumption can induce intracellular hypoxia and activate HIF. Depending on its intensity, this response could either play an important role in beta-cell adaptation to increased insulin demand under physiological conditions (physiological hypoxia) or be involved in the detrimental effect of chronic hyperglycemia.

Earlier studies have convincingly shown that glucose rapidly increases O_2_ consumption in islets from rats [8], [15], [27], mice [12], non-human primates and humans [9]. Although we did not repeat these measurements of islet O_2_ consumption or intra-islet pO_2_, we have shown that glucose increases the formation of pimonidazole-protein adducts in islets and INS-1E cells in parallel with changes in HIF-target gene expression and HIF nuclear accumulation. Moreover, these effects were inhibited under hyperoxic conditions. Pimonidazole-protein adducts were not more abundant in the center than at the islet periphery, indicating that our results do not simply result from central necrosis. In addition, this staining was heterogeneous between individual cells, as would be expected from the metabolic heterogeneity of beta-cells [28].

The increase by glucose of HIF1α nuclear levels in rat beta-cells is in good agreement with the fact that glucose induces moderate hypoxia and with current and previous observations that hypoxia activates HIF1 in INS-1E cells and cultured islets [14], [23], [29]. It is also compatible with the recent report that *Hif1α* gene inactivation corrected the stimulation of HIF-target gene expression following *vhlh* inactivation in mouse beta-cells [30]. In contrast, the increase of HIF2α nuclear levels in INS-1E cells by glucose, CoCl_2_ and hypoxia is surprising because HIF2α was detected neither in hypoxic embryonic pancreatic explants [31] nor in *vhlh*-knockout mouse islets [30]. However, the role of HIF2α in INS-1E cells is strongly supported by the observation that *Hif2α* expression is absolutely required for the stimulation of *Adm* mRNA expression. Of note, glucose, CoCl_2_ and hypoxia also affected *Hifα* mRNA levels in rat beta-cells through unknown mechanisms, but the relative contribution of these changes to the global increase in HIF activity and HIF-target gene expression has not been investigated. In any case, these changes in *Hifα* mRNA levels should only modulate the increase in HIFα protein levels that mainly result from their stabilization under hypoxic conditions [1].

It is well established that glucose stimulates the expression of various glycolytic enzymes in cultured insulin-secreting cells and rodent islets, including GK, GAPDH and liver pyruvate kinase [32], [33]. It has been shown that the stimulation of expression of liver pyruvate kinase results from a reduction in AMPK activity [34] and from activation of the transcription factors ChREBP and c-MYC [35], [36]. In contrast, the stimulation of GK expression has been ascribed to SREBP1c activation [37]. However, the transcription factors involved in the glucose-induced expression of other glycolytic enzymes in beta-cells are poorly characterized. In other tissues, HIF1α is preferentially involved in the regulation of glycolytic enzymes while HIF2α stimulates the expression of genes related to angiogenesis [38]. In INS-1E cells, knockdown of *Hif1α* and *Hif2α* had a stronger effect on the glucose stimulation of *Tpi1* and *Gapdh* than knockdown of either isoform alone. This suggests that both HIF1 and HIF2 modulate the expression of glycolytic enzymes, at least in rat beta-cells. However, neither hyperoxia, nor *Hif1α/Hif2α* knockdown or inhibition of Ca^2+^ influx with nimodipine were able to fully inhibit the glucose induction of glycolytic enzymes (except for the complete inhibition of *Ldha* mRNA by hyperoxia in whole rat islets), confirming that other transcription factors, e.g. *Myc* [39], are also involved. This remark is particularly important in the case of *Gapdh* mRNA, the glucose induction of which was unaffected by hyperoxia and nimodipine treatment. On the other hand, hyperoxia, *Hif2α* but not *Hif1α*) knockdown and nimodipine almost fully inhibited the expression of *Adm*, thereby demonstrating the specific role of HIF2 in *Adm* expression by glucose-induced decrease in islet pO_2_. Thus, both HIF isoforms are not redundant and play distinct roles in beta-cell gene expression.

### Possible relevance for the physiology and pathophysiology of beta-cells

High expression of GK and downstream glycolytic enzymes is critical for GSIS [40], [41]. In agreement, global down-regulation of glycolytic enzymes in *Hif1α* or *Arnt* knockout beta-cells markedly reduced GSIS and *in vivo* glucose tolerance in some [42], [43] but not all studies [22], [30]. In siRNA-treated INS-1E cells, knockdown of *Hif1α* markedly inhibited GSIS during culture while the inhibition of *Hif2α* tended to increase basal insulin release, suggesting that the absence of each isoform differently affects beta cell function. In this context, it is important to note that the moderate activation of HIF by glucose stimulation under physiological conditions, i.e. *in vivo* where the islet pO_2_ may be lower than in *in vitro* culture systems, could play an important role in the maintenance of the beta-cell phenotype or in their adaptation to changes in insulin demand. Such “physiological hypoxia” is compatible with the fact that HIF is activated at glucose concentrations at which mitochondrial ATP production is not reduced [44]. In support of this hypothesis, it was recently shown that, in the rat, the proportion of islets showing pimonidazole-protein adduct staining *in vivo* is modulated by changes in insulin demand [45]. In contrast with that study, however, we did not observe major differences in pimonidazole staining between islets from the same mouse pancreatic section.

On the other hand, almost complete repression of low-*K*_m_ HK (I–III), LDH and MCT (isoforms 1, 2 and 4) is also critical to prevent inappropriate stimulation of insulin secretion at low glucose and during exercise [41], [46]. The lack of LDH and high expression of malate/aspartate and glycerol-phosphate shuttle enzymes also contribute to optimal GSIS by preventing pyruvate diversion from its mitochondrial metabolism while coupling re-oxidation of cytosolic NADH with increased mitochondrial ATP production [47]. Following *vhlh* inactivation in beta-cells, sustained activation of HIF-target gene expression under normoxic conditions tended to increase insulin secretion at low glucose while reducing the maximal GSIS with consequent development of glucose intolerance [22], [30], [48]. Moreover, expression of a constitutively active form of HIF1α significantly decreased GSIS, O_2_ consumption and pimonidazole staining in MIN6 cells [16]. Hypoxia should therefore be considered as a possible contributing factor when interpreting the *in vitro* effects of high glucose concentrations on beta-cell gene expression, function and survival, even in beta-cell lines. Our recent attempt to prevent beta-cell glucotoxicity by culturing islets for 1 week in the presence of 60% O_2_ led to the opposite effect, i.e. a ∼10-fold increase in islet cell apoptosis in G10 and a ∼50-fold increase in G30 (Figure S4). Other strategies will have to be developed to check the role of hypoxia in beta-cell glucotoxicity.

### Possible relevance for the pathophysiology of type 2 diabetes

Together with alterations of islet microvasculature and fibrosis that impair vessel integrity and O_2_ supply [20], the high metabolic demand imposed by hyperglycaemia may promote beta-cell hypoxia and HIF activation *in vivo*. So far, the available evidence suggests, but do not prove, that some beta-cells may indeed suffer from hypoxia in type 2 diabetes. Thus, HIF-target gene expression (this study and [18]–[20]) and pimonidazole-protein adducts [16] were increased in islets isolated from various rodent models of diabetes, with a few islet cells (the identity of which could not be clarified) displaying higher levels of pimonidazole-protein adducts when measured by immunohistochemistry. Moreover, HIF1α-positive islet nuclei, although rare, were clearly detected on pancreatic sections of diabetic but not normoglycaemic mice. If these cells were unambiguously identified as hypoxic beta-cells, one could suggest that *in vivo* hypoxia, HIF activation and HIF-target gene expression in only a fraction of beta-cells could contribute to the slow deterioration of beta-cell function and survival in type 2 diabetes. Such *in vivo* hypoxia could result from increased beta-cell O_2_ consumption at high glucose, to a decrease in islet perfusion following changes in islet microvasculature by chronic hyperglycemia, or to both processes.

In conclusion, glucose-induced O_2_ consumption creates an intracellular hypoxia that activates HIF1 and HIF2 in rat beta-cells, and this glucose effect contributes, together with the activation of other transcription factors, to the glucose stimulation of expression of some glycolytic enzymes and other hypoxia response genes.

## Materials and Methods

### Reagents

Succinic acid monomethyl ester, α-ketoisocaproate, diazoxide, nimodipine and 3-O-methyl-D-glucopyranose were purchased from Sigma (St. Louis, MO, USA). DharmaFECT1 and siGENOME SMARTpool small interfering RNA (siRNA) duplexes targeting *Hif1α* and *Hif2α* were from Dharmacon (Thermo Scientific, Lafayette, CO, USA). Silencer® Firefly Luciferase (*Luc*) and Negative Control siRNA were from Ambion (Applied Biosystems, Foster City, CA, USA). Other reagents were from Merck (Darmstadt, Germany).

### Antibodies

Rabbit polyclonal anti-Actin antibody (A2066) was from Sigma. Mouse monoclonal anti-HIF1α (ab1) and anti-HIF2α (ab8365) antibodies used for western blotting were from Abcam (Cambridge, UK). Mouse anti-HIF1β antibody (clone 29, 611079) was from BD Biosciences (San Jose, CA, USA) and rabbit anti-HIF1β/ARNT antibody (3718) was from Cell Signaling Technology (Danvers, MA, USA). The rabbit anti-HIF1α antiserum used for immunohistochemistry has been described earlier [49].

### Islet isolation and culture

Pancreatic islets were isolated from ∼200 g male Wistar rats as described [50]. They were precultured for 1 week in serum-free RPMI 1640 medium (Invitrogen, Carlsbad, CA, USA) containing 10 mmol/l glucose, 5 g/l BSA (fraction V, Roche, Basel, Switzerland), 100 IU/ml penicillin and 100 µg/ml streptomycin (Invitrogen). Islets that developed central necrosis were discarded during preculture. After preculture, islets were cultured 18 h in the G2, G5, G10, and G30 and various test substances at different incubator pO_2_ (38, 151, 456 and 680 mmHg). After culture, the medium was collected for insulin concentration determination (RIA using rat insulin as a standard) and the islets were processed for further analysis.

### *In vivo* studies

*Lepr^db/db^* and *Lepr^db/+^* mice on a C57BL/KSJ background were from Janvier (Le Genest-Saint-Isle, France) or from the animal facility of the Garvan Institute. They were used from 10 to 26 weeks of age. Blood glucose was measured with a glucometer (Accu-Check Sensor, Roche, Mannheim, Germany). After cervical dislocation, the duodenal loop, the pancreas and the spleen were removed as a block and fixed in less than 3 minutes. All experiments were approved by the local ethics committee for animal experimentation (Université catholique de Louvain, Faculté de Médecine, Comité d'éthique facultaire pour l'expérimentation animale, projet UCL/MD/2009/009: “Mécanismes moléculaires de la plasticité du phénotype des cellules B pancréatiques en conditions physiopathologiques” accepted for 4 years). “Principles of laboratory animal care” (NIH publication no. 85–23, revised 1985) were followed.

### Cell culture and RNA interference

INS-1E cells (passage 70–94) were cultured in standard RPMI medium supplemented with 10% heat-inactivated fetal calf serum, 10 mmol/l HEPES, 1 mmol/l sodium pyruvate, 2 mmol/l glutamine, 100 U/ml penicillin, 100 µg/ml streptomycin, and 50 µmol/l β-mercaptoethanol. To downregulate gene expression, INS-1E cells were transfected for 24 h using DharmaFECT1 and 30 nmol/l siRNA in antibiotic-free medium, according to the manufacturer's instructions. Cells (∼70%confluence) were then cultured for 18 h in fresh medium containing different glucose concentration and various test substances as indicated.

### Real time RT-PCR

Islet and INS-1E total RNA extraction, reverse transcription, real-time PCR and melting curve analysis of PCR products were performed as described previously [50]. Relative changes in *Gene* to *Tbp* mRNA ratio between test and control conditions were computed using the 2^−ΔΔCt^ method. Primer sequences and reaction conditions are detailed in Tables S2 and S3.

### Nuclear extraction and western blotting for HIF1α, HIF2α and ARNT

INS-1E cells were rinsed with ice-cold PBS, scraped in hypotonic buffer (20 mmol/l HEPES, 5.7 mmol/l NaF, 1 mmol/l EDTA, pH 7.5) and incubated on ice for 15 min. After addition of 0.5% Nonidet P-40, the nuclei were pelleted (800×g for 4 min at 4°C), resuspended in 50 µl Complete Lysis Buffer AM1 (Active Motif, Rixensart, Belgium) supplemented with 1 mmol/l dithiothreitol and 1% protease inhibitor cocktail (Roche Diagnostics, Mannheim, Germany), incubated for 15 min on ice with regular shaking, and centrifuged at 1750×g for 10 min at 4°C. Cytosolic proteins in the supernatant were precipitated with trichloroacetic acid, triple extracted with ether, and solubilised in Laemmli buffer. Nuclear and cytosolic extracts were then separated by 7.5% SDS-PAGE and transferred to nitrocellulose membrane (Bio-Rad Laboratories, Hercules, CA, USA). The membranes were incubated with a mouse monoclonal primary antibody followed by a horse-radish-peroxidase-conjugated anti-mouse antibody (Santa-Cruz, CA, USA) and the signal was revealed by enhanced chemiluminescence (SuperSignal® West-Femto or West-Dura kits, Thermo Scientific). Band intensities were quantified by scanning densitometry (Gel-Doc2000, Bio-Rad), analyzed with Quantity One™ (Bio-Rad) and normalized to Red Ponceau staining or ACTIN band intensity.

### Immunodetection of HIF1α and ARNT

Tissues were fixed in 4% formaldehyde and embedded in paraffin. For HIF1α and insulin detection, 5 µm-thick sections were treated as described previously [31]. For ARNT detection, the sections were incubated with rabbit anti-ARNT antibody diluted 1∶50 overnight at 4°C, washed in Tris-buffered saline and incubated for 1 h with Alexa Red 594-conjugated goat anti-rabbit IgG (Molecular Probes) diluted 1∶100. After washing in Tris-buffered saline and addition of Vectashield-mounting medium containing DAPI (Vector Laboratories, Burlingame, CA), sections were visualized on an Axioskop 40 microscope (Zeiss, Oberkochen, Germany) equipped with an Infinity-x camera (Deltapix, Lumenera Corporation, Ottawa, Ontario, Canada).

### Immunodetection of pimonidazole-protein adducts

Pimonidazole (Hypoxyprobe™ Inc., Burlington, MA, USA) was added to RPMI medium at a final concentration of 200 µmol/l 2 h before the end of culture or was injected intraperitoneally (60 mg/kg body weight, 34 mmol/l in NaCl 9 g/l sterile solution) 24 h before killing the mice. Tissues were fixed in 4% formaldehyde and embedded in paraffin before detection of pimonidazole-protein adducts on 5 µm-thick sections. Briefly, deparaffinized sections were treated with H_2_O_2_ (0.3% vol/vol) to inactivate endogenous peroxidase and incubated with either Hypoxyprobe 1 monoclonal antibody (clone 4.3.11.3) diluted 1∶100 followed by anti-mouse EnVision+™ peroxidase complex for 1 h (Dako, Carpintera, USA) for rat islets, or Hypoxyprobe 1 polyclonal rabbit antibody diluted 1∶200 followed by anti-rabbit EnVision+™ peroxidase complex for mouse pancreas. In both cases, the signal was revealed by 3,3′-diaminobenzidine. Pimonidazole-protein adducts in cultured islets were also measured by Western Blot and the signal intensity was normalized to that of ACTIN (Ab dilution 1∶2000).

### Statistical analysis

Results are means ± SEM for the indicated number of experiments. Statistical significance of differences between groups was assessed by one-way ANOVA and a test of Newman-Keuls or by two-way ANOVA and a test of Bonferroni. Differences were considered significant when *p*<0.05.

## Supporting Information

Figure S1 **Effects of glucose, hypoxia and CoCl_2_ on ARNT (HIF1β) protein levels in cultured rat islets.** The islets were cultured exactly as described in legend to figure 1. ARNT and insulin were detected by immunohistochemistry in 5 µm-thick islet sections. Nuclei were stained with 0.75 µg/ml 4′,6-diamidino-2-phenylindole (DAPI). Results are representative for 2 to 3 experiments.
(TIF)

Figure S2 **Role of hypoxia in glucose-induced HIF-target gene mRNA expression in cultured rat islets.** One week precultured islets were cultured 18 h in G2, G5, G10 or G30 in the presence of 20% O_2_ (pO_2_∼151 mmHg, open circles) or 90% O_2_ (pO_2_∼680 mmHg, close circles). Pimonidazole was added to the culture medium for the last 2 h and pimonidazole protein-adducts were detected by western blot. For each lane, the area under the curve (AUC) was calculated and normalized for changes in ACTIN band intensity. Results are representative blots and means ± SEM of normalized AUC for 3 experiments. *, p<0.05 for the effect of glucose *vs.* G2 and ^#^, *p<0.05* for the effect of 90% O_2_ (two-way ANOVA+test of Bonferroni).
(TIF)

Figure S3 **Role of Ca^2+^ influx and insulin secretion in glucose-induced HIF-target gene expression in INS-1E cells.** INS-1E cells (70% of confluence) were cultured 18 h in various glucose concentrations in the presence (closed circles) or absence (open circles) of 1 µmol/l nimodipine. Gene to Tbp mRNA levels were expressed relative to the level in G2. Data are means ± SEM for 3 to 4 experiments. *, p<0.05 for the effect of glucose, #, p<0.05 for the effect of nimodipine, §, p<0.05 for the effect of insulin (two-way ANOVA+test of Bonferroni).
(TIF)

Figure S4 **Effects of a one-week culture under hyperoxic conditions on islet cell DNA fragmentation.** Rat islets were cultured for 1 week in the presence of G10 or G30 under normoxic or hyperoxic conditions, as detailed in legend to figure 6D. At the end of culture, islet DNA fragmentation was assessed with the Cell Death ELISA kit from Roche, as described in reference 21. Results are mean and individual data for 2 independent cultures.
(TIF)

Table S1 **Rat islet mRNA expression of genes involved in HIF signaling pathway and their regulation by glucose.** After 1 week preculture in serum-free RPMI medium containing 5 g/l BSA and 10 mmol/l glucose (G10), rat islets were cultured 18 h in the presence of 2, 5, 10 or 30 mmol/l glucose. The glucose regulation of gene mRNA levels was measured using Affymetrix rat 230.2 microarrays (for details, see [21]). Probe-sets corresponding to various components of the HIF signaling pathway were selected based on the literature [1],[51] and classified in three groups: Transcription factors, HIF-regulating and interacting proteins, and HIF-target genes. *, # denotes genes whose expression was up-regulated at least 1.5-fold (*) or more than 2-fold (#) in *vhlh*-KO *vs.* WT mouse islets [22]. Data are means ± SE hybridization value (Arbitrary units) for 4 experiments. ^a^ *P*<0.05, ^b^ *P*<0.01 *vs.* islets cultured in G2 (one-way ANOVA+test of Newman-Keuls). The following probe sets were considered «Absent» on the microarrays (for analysis criteria, see [21]): *Adra1b* (1368574_at); *# Abcb1* (1370465_at); * Abcg2 (1380577_at); *Col5a1* (1369955_at); *Cp* (1368419_at; 1368420_at); * Ctgf (1367631_at); *Cxcl12* (1387655_at; 1388583_at); *Cyp2s1* (1390282_at); *Edn1* (1369519_at); * Egln1 (1375262_at); *Eng* (1372579_at); *Epo* (1387308_at); *Hif1α* (1368149_at); * Hk1 (1386929_at); * Hk2 (1369006_at; 1383519_at);*Igf2* (1371206_a_at; 1398322_at); *Igfbp3* (1386881_at); *Itgb2* (1383131_at); * Krt14 (1371895_at); *Lep* (1387748_at); *Lox* (1368171_at; 1368172_a_at); * Lrp1 (1388416_at); *Mmp14* (1378225_at); *Mmp2* (1369825_at); *Nos2* (1387667_at); *Nos3* (1371166_at); *# Pfkfb3* (1369794_a_at; 1397082_at); *Pgk1* (1368906_at); *Plaur* (1387269_s_at); *Prok1* (1387650_at); *# Serpine1* (1368519_at; 1392264_s_at); *Slc2a3* (1372326_at; 1387707_at); *Tert* (1388222_at); *Tf* (1370228_at; 1391323_at); *Tgfb3* (1367859_at); *# Tgm2* (1387776_at).
(DOC)

Table S2 **Sequences of oligonucleotide primers and reaction conditions for real-time PCR amplification of rat cDNA, and characteristics of PCR products.** Tm: Amplicon melting temperature; ^a^: Islet sample cDNA quantity per tube (ng total RNA equivalent).
(DOC)

Table S3 **Sequences of oligonucleotide primers for real-time PCR amplification of mouse cDNA.**
(DOC)
