# Supplementary figures and images for: Glucose-Induced O2 Consumption Activates Hypoxia Inducible Factors 1 and 2 in Rat Insulin-Secreting Pancreatic Beta-Cells
Source: PLoS One. 2012 Jan 3;7(1):e29807. doi: 10.1371/journal.pone.0029807 (PMC3250482; doi:10.1371/journal.pone.0029807)

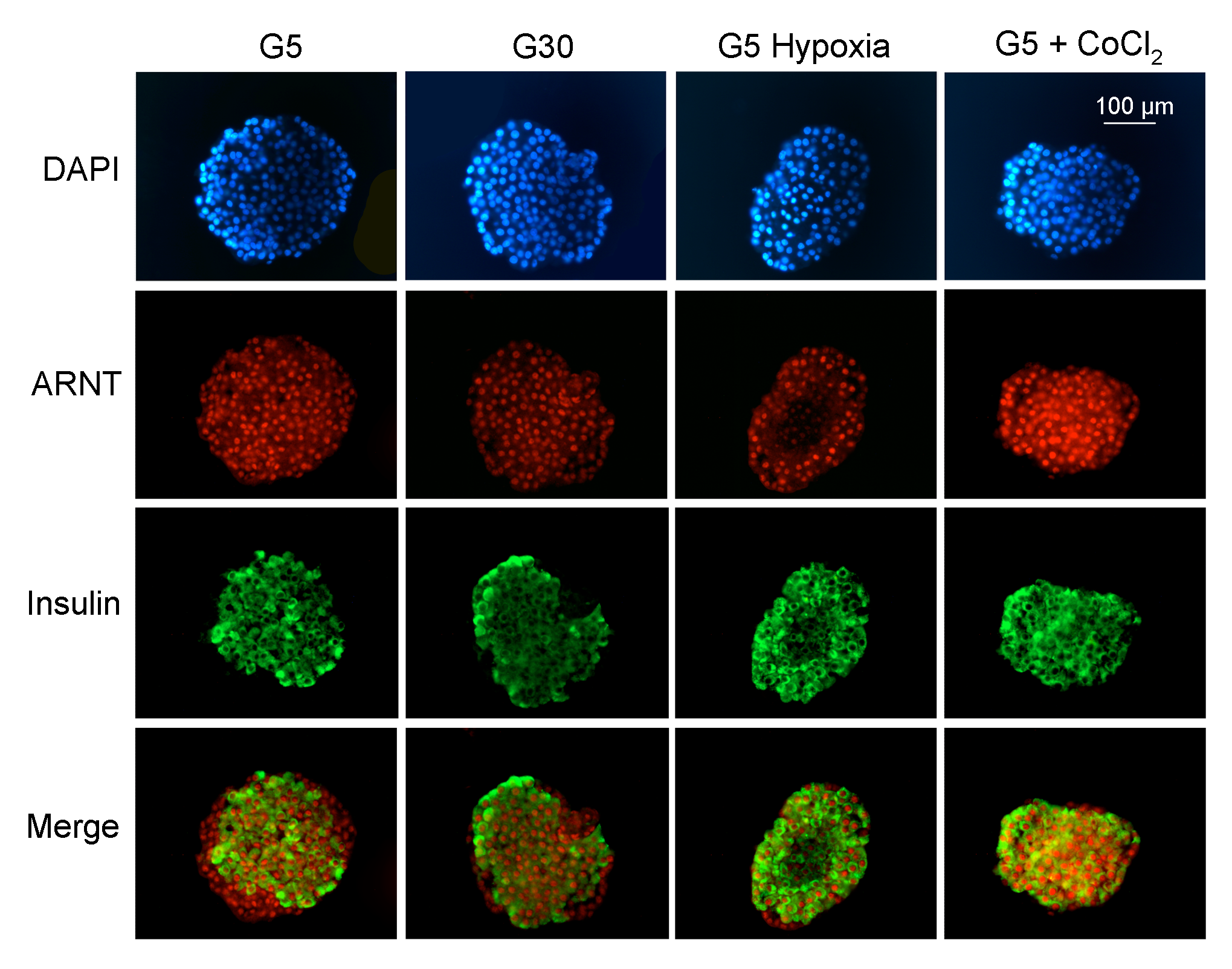

Supplement: Figure S1 — Effects of glucose, hypoxia and CoCl2 on ARNT (HIF1β) protein levels in cultured rat islets. The islets were cultured exactly as described in legend to figure 1. ARNT and insulin were detected by immunohistochemistry in 5 µm-thick islet sections. Nuclei were stained with 0.75 µg/ml 4′,6-diamidino-2-phenylindole (DAPI). Results are representative for 2 to 3 experiments. (TIF) [file pone.0029807.s001.tif]

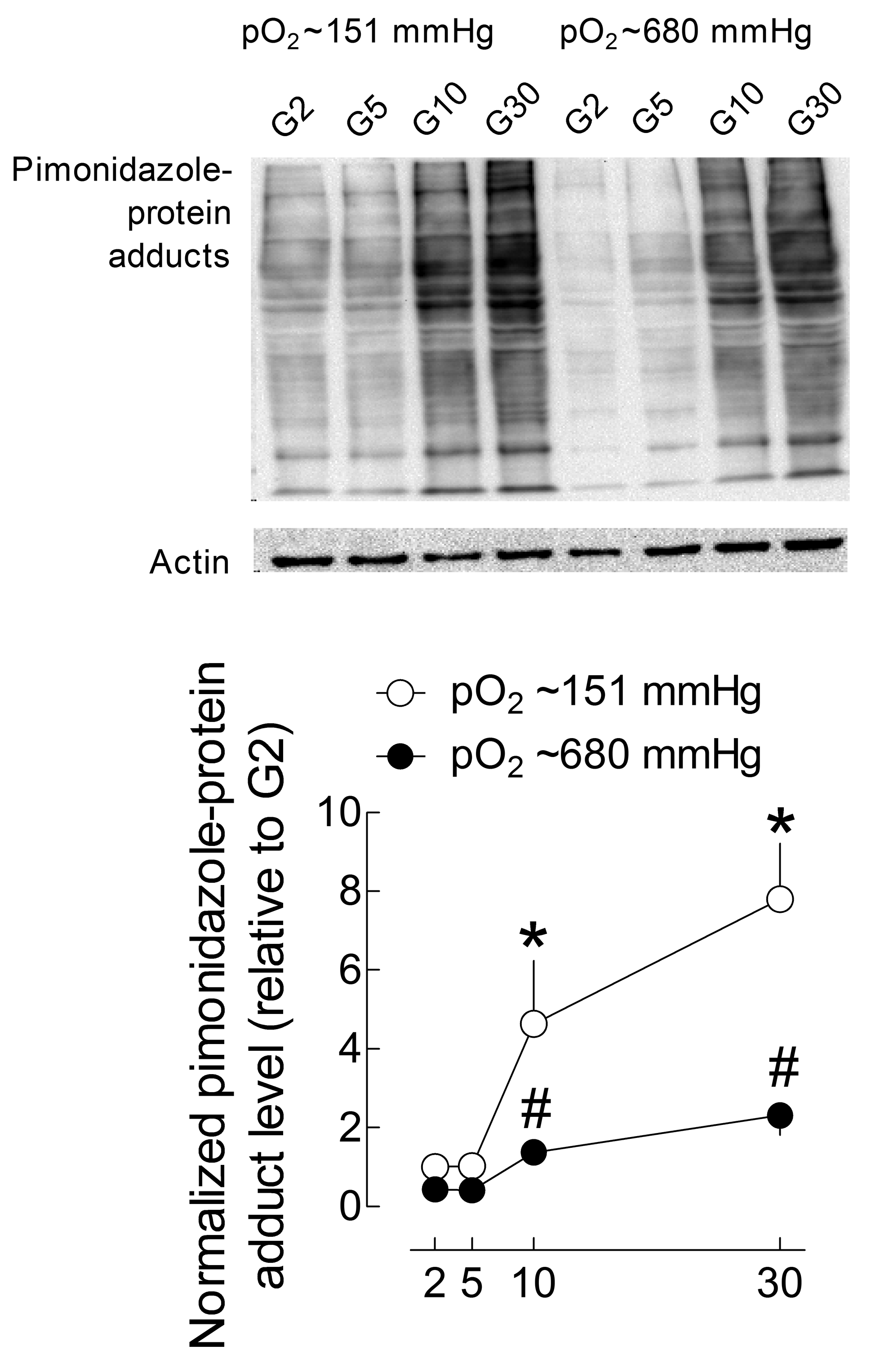

Supplement: Figure S2 — Role of hypoxia in glucose-induced HIF-target gene mRNA expression in cultured rat islets. One week precultured islets were cultured 18 h in G2, G5, G10 or G30 in the presence of 20% O2 (pO2∼151 mmHg, open circles) or 90% O2 (pO2∼680 mmHg, close circles). Pimonidazole was added to the culture medium for the last 2 h and pimonidazole protein-adducts were detected by western blot. For each lane, the area under the curve (AUC) was calculated and normalized for changes in ACTIN band intensity. Results are representative blots and means ± SEM of normalized AUC for 3 experiments. *, p<0.05 for the effect of glucose vs. G2 and #, p<0.05 for the effect of 90% O2 (two-way ANOVA+test of Bonferroni). (TIF) [file pone.0029807.s002.tif]

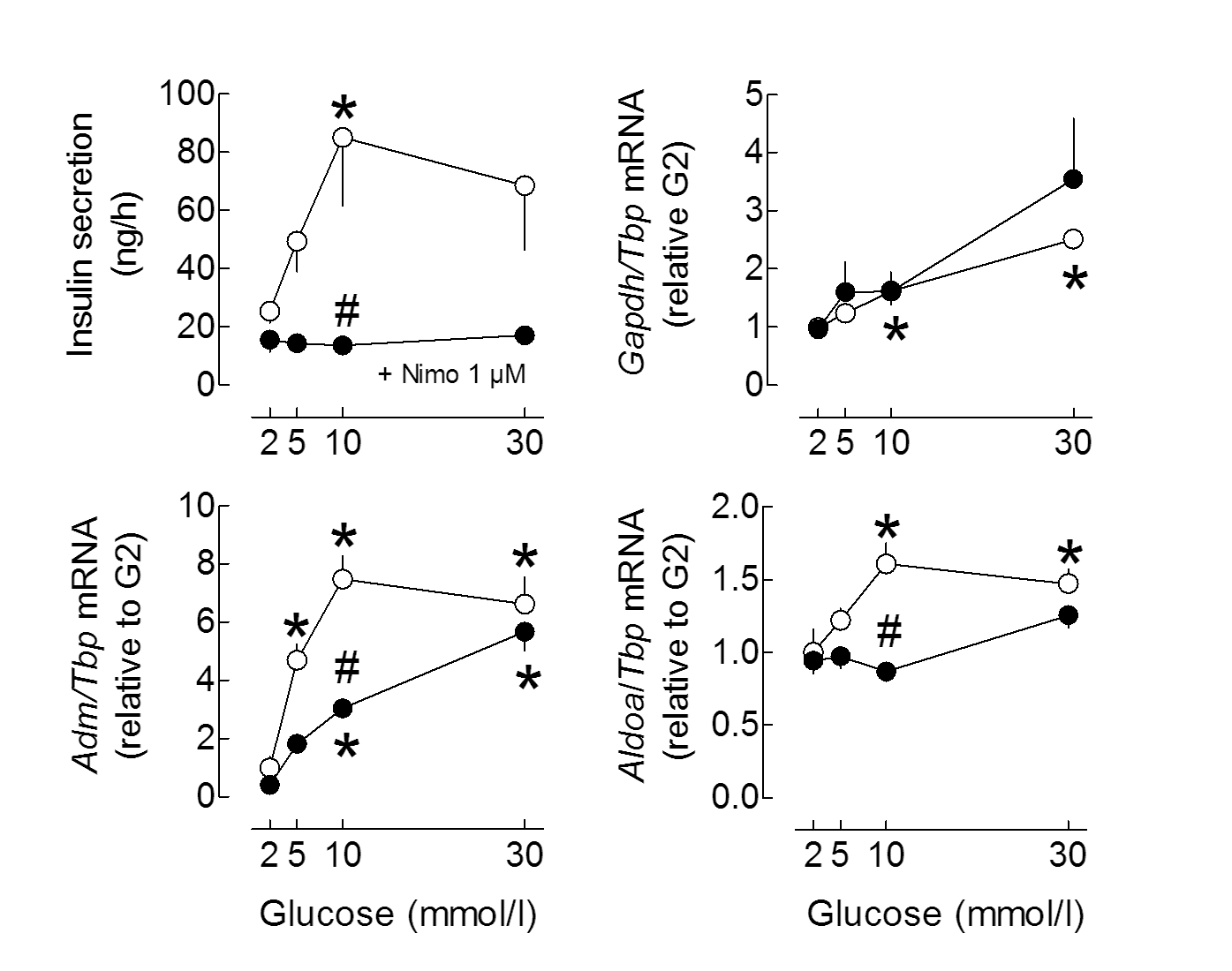

Supplement: Figure S3 — Role of Ca2+ influx and insulin secretion in glucose-induced HIF-target gene expression in INS-1E cells. INS-1E cells (70% of confluence) were cultured 18 h in various glucose concentrations in the presence (closed circles) or absence (open circles) of 1 µmol/l nimodipine. Gene to Tbp mRNA levels were expressed relative to the level in G2. Data are means ± SEM for 3 to 4 experiments. *, p<0.05 for the effect of glucose, #, p<0.05 for the effect of nimodipine, §, p<0.05 for the effect of insulin (two-way ANOVA+test of Bonferroni). (TIF) [file pone.0029807.s003.tif]

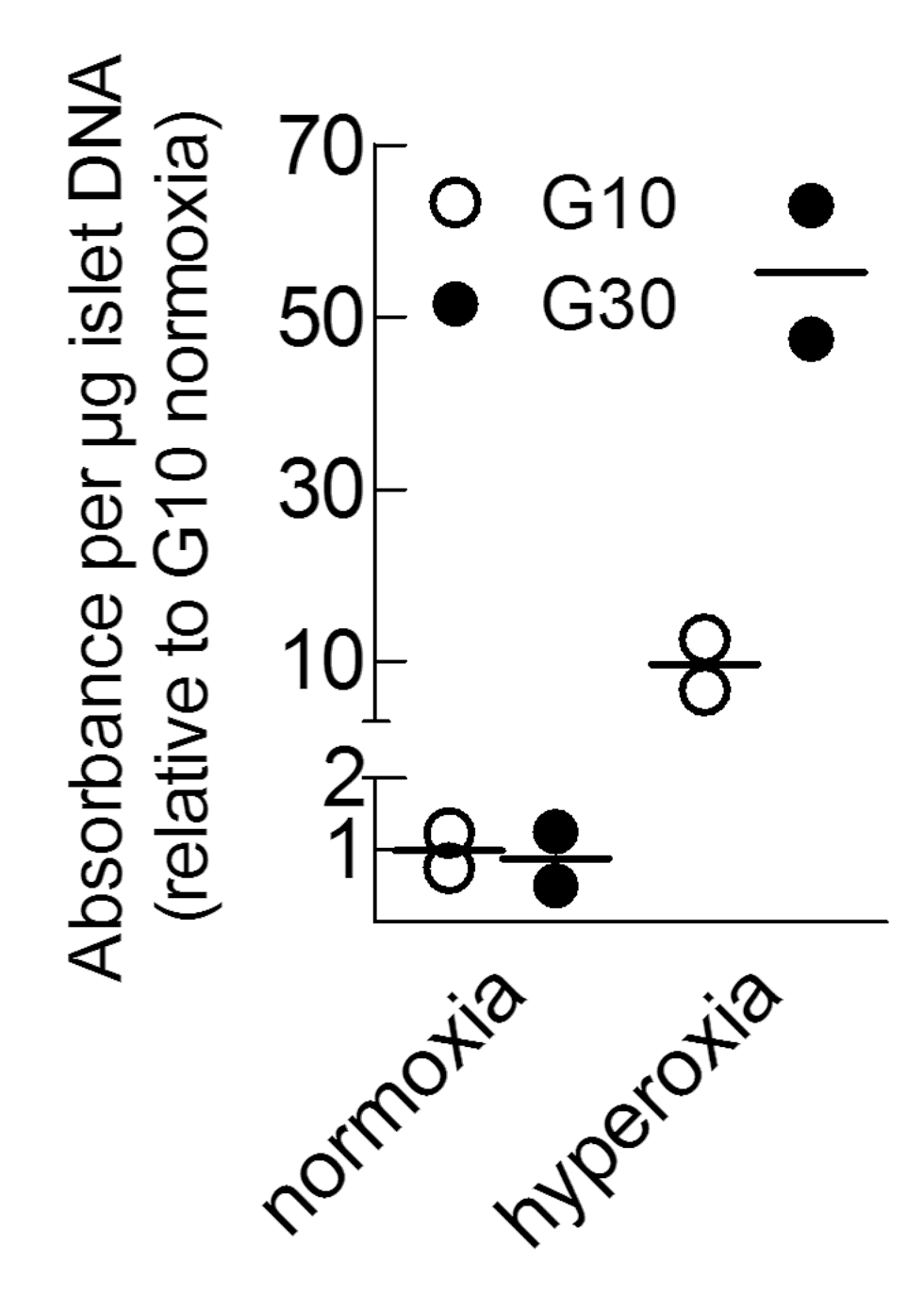

Supplement: Figure S4 — Effects of a one-week culture under hyperoxic conditions on islet cell DNA fragmentation. Rat islets were cultured for 1 week in the presence of G10 or G30 under normoxic or hyperoxic conditions, as detailed in legend to figure 6D. At the end of culture, islet DNA fragmentation was assessed with the Cell Death ELISA kit from Roche, as described in reference 21. Results are mean and individual data for 2 independent cultures. (TIF) [file pone.0029807.s004.tif]
