# Supplementary material for: Glucose-Induced O2 Consumption Activates Hypoxia Inducible Factors 1 and 2 in Rat Insulin-Secreting Pancreatic Beta-Cells
Source: PLoS One. 2012 Jan 3;7(1):e29807. doi: 10.1371/journal.pone.0029807 (PMC3250482; doi:10.1371/journal.pone.0029807)
Supplement: Table S1 — Rat islet mRNA expression of genes involved in HIF signaling pathway and their regulation by glucose. After 1 week preculture in serum-free RPMI medium containing 5 g/l BSA and 10 mmol/l glucose (G10), rat islets were cultured 18 h in the presence of 2, 5, 10 or 30 mmol/l glucose. The glucose regulation of gene mRNA levels was measured using Affymetrix rat 230.2 microarrays (for details, see [21]). Probe-sets corresponding to various components of the HIF signaling pathway were selected based on the literature [1],[51] and classified in three groups: Transcription factors, HIF-regulating and interacting proteins, and HIF-target genes. *, # denotes genes whose expression was up-regulated at least 1.5-fold (*) or more than 2-fold (#) in vhlh-KO vs. WT mouse islets [22]. Data are means ± SE hybridization value (Arbitrary units) for 4 experiments. a P<0.05, b P<0.01 vs. islets cultured in G2 (one-way ANOVA+test of Newman-Keuls). The following probe sets were considered «Absent» on the microarrays (for analysis criteria, see [21]): Adra1b (1368574_at); # Abcb1 (1370465_at); * Abcg2 (1380577_at); Col5a1 (1369955_at); Cp (1368419_at; 1368420_at); * Ctgf (1367631_at); Cxcl12 (1387655_at; 1388583_at); Cyp2s1 (1390282_at); Edn1 (1369519_at); * Egln1 (1375262_at); Eng (1372579_at); Epo (1387308_at); Hif1α (1368149_at); * Hk1 (1386929_at); * Hk2 (1369006_at; 1383519_at);Igf2 (1371206_a_at; 1398322_at); Igfbp3 (1386881_at); Itgb2 (1383131_at); * Krt14 (1371895_at); Lep (1387748_at); Lox (1368171_at; 1368172_a_at); * Lrp1 (1388416_at); Mmp14 (1378225_at); Mmp2 (1369825_at); Nos2 (1387667_at); Nos3 (1371166_at); # Pfkfb3 (1369794_a_at; 1397082_at); Pgk1 (1368906_at); Plaur (1387269_s_at); Prok1 (1387650_at); # Serpine1 (1368519_at; 1392264_s_at); Slc2a3 (1372326_at; 1387707_at); Tert (1388222_at); Tf (1370228_at; 1391323_at); Tgfb3 (1367859_at); # Tgm2 (1387776_at). (DOC) [file pone.0029807.s005.doc]

| *Gene symbol* (probe-set ID) | **G2** | **G5** | **G10** | **G30** |
| --- | --- | --- | --- | --- |
| HIF subunits |  |  |  |  |
| *Hif1α* (1387076_at) | 2091 ± 34 | 1863 ± 51 ^b^ | 1489 ± 23 ^b^ | 1262 ± 7 ^b^ |
| *Hif2α* (1369703_at) | 23 ± 6 | 34 ± 6 | 53 ± 11 | 54 ± 10 |
| *Arnt (Hif1β)* (1369244_at) | 82 ± 6 | 65 ± 6 | 62 ± 8 | 60 ± 1 |
| HIF1β-related transcription factors |  |  |  |  |
| *Arntl* (1370510_a_at) | 102 ± 11 | 82 ± 8 | 114 ± 6 | 144 ± 8 ^a^ |
| *Arnt2* (1373315_at) | 376 ± 25 | 419 ± 12 | 253 ± 10 ^b^ | 227 ± 13 ^b^ |
| *Arnt2* (1370611_at) | 55 ± 6 | 47 ± 5 | 49 ± 6 | 36 ± 3 |
| HIF-regulating and interacting proteins |  |  |  |  |
| ** Egln1 (Phd2)* (1389207_at) | 187 ± 10 | 218 ± 8 | 193 ± 16 | 172 ± 12 |
| ** Egln2 (Phd1)* (1373794_at) | 388 ± 16 | 399 ± 35 | 387 ± 30 | 348 ± 35 |
| *# Egln3 (Phd3)* (1368174_at) | 91 ± 7 | 85 ± 6 | 59 ± 3 ^a^ | 86 ± 7 |
| *Ard1* (1383922_a_at) | 45 ± 11 | 61 ± 7 | 61 ± 12 | 72 ± 12 |
| *Ard1* (1384340_a_at) | 69 ± 7 | 83 ± 6 | 96 ± 6 | 93 ± 9 |
| *Vhl* (1368076_at) | 452 ± 10 | 393 ± 13 ^b^ | 291 ± 8 ^b^ | 297 ± 8 ^b^ |
| HIF-target genes |  |  |  |  |
| Glucose metabolism: |  |  |  |  |
| *# Slc2a1* (1370848_at) | 211 ± 13 | 166 ± 15 ^a^ | 176 ± 5 ^a^ | 234 ± 6 |
| *# Gpi* (1371392_at) | 592 ± 29 | 608 ± 19 | 689 ± 25 | 1091 ± 58 ^b^ |
| *# Pfkl* (1367743_at) | 78 ± 3 | 74 ± 11 | 88 ± 8 | 167 ± 5 ^b^ |
| *# Pfkp* (1372182_at) | 276 ± 20 | 273 ± 9 | 206 ± 18 ^a^ | 458 ± 22 ^b^ |
| *# Aldoa* (1367617_at) | 2766 ± 37 | 3374 ± 100 ^a^ | 3706 ± 113 ^b^ | 4899 ± 251 ^b^ |
| *# Aldoc* (1386998_at) | 65 ± 9 | 66 ± 9 | 95 ± 7 | 74 ± 7 ^b^ |
| *# Tpi1* (1367603_at) | 868 ± 45 | 1048 ± 52 | 1450 ± 39 ^a^ | 3340 ± 233 ^b^ |
| *# Gapdh* (1367557_s_at) | 2605 ± 118 | 2945 ± 83 | 4156 ± 102 ^b^ | 6905 ± 353 ^b^ |
| *# Pgk1*(1387361_s_at) | 704 ± 27 | 801 ± 24 | 792 ± 28 | 1433 ± 37 ^b^ |
| *# Pgk1*(1388318_at) | 1520 ± 57 | 1820 ± 88 | 1773 ± 89 | 3148 ± 208 ^b^ |
| *# Pgam1*(1386864_at) | 1959 ± 54 | 2191 ± 42 | 2879 ± 46 ^b^ | 3911 ± 155 ^b^ |
| *# Eno1* (1367575_at) | 911 ± 51 | 964 ± 69 | 1133 ± 51 | 2834 ± 161 ^b^ |
| *Pkm2* (1369931_at) | 2285 ± 59 | 2629 ± 69 ^a^ | 2920 ± 43 ^a^ | 5279 ± 280 ^b^ |
| *Pklr* (1368651_at) | 139 ± 10 | 133 ± 6 | 168 ± 18 | 290 ± 29 ^b^ |
| *Pklr* (1387263_at) | 41 ± 2 | 65 ± 3 | 101 ± 10 ^b^ | 177 ± 14 ^b^ |
| *# Ldha* (1367586_at) | 78 ± 6 | 76 ± 15 | 102 ± 19 | 171 ± 27 ^a^ |
| *# Pdk1* (1368079_at) | 122 ± 10 | 132 ± 14 | 102 ± 12 | 273 ± 23 ^b^ |
| Vascular tone: |  |  |  |  |
| *# Adm* (1387219_at) | 56 ± 8 | 51 ± 7 | 28 ± 4 | 284 ± 51 ^a^ |
| Angiogenesis: |  |  |  |  |
| *Vegfa* (1373807_at) | 1977 ± 43 | 2174 ± 53 | 1964 ± 71 | 1957 ± 58 |
| *Vegfa* (1370081_a_at) | 818 ± 55 | 847 ± 57 | 714 ± 25 | 798 ± 44 |
| *Vegfb* (1380854_at) | 142 ± 8 | 151 ± 14 | 137 ± 7 | 106 ± 14 |
| ** Vegfc* (1368463_at) | 88 ± 9 | 78 ± 7 | 60 ± 6 ^a^ | 70 ± 2 |
| pH regulation: |  |  |  |  |
| *Car9* (1393452_at) | 23 ± 3 | 30 ± 3 | 57 ± 7 ^b^ | 45 ± 8 ^a^ |
| *# Car12* (1371922_at) | 24 ± 3 | 27 ± 2 | 22 ± 6 | 134 ± 39 ^b^ |
| Cell cycle regulation: |  |  |  |  |
| ** Ccng2* (1371953_at) | 363 ± 28 | 339 ± 18 | 257 ± 14 ^b^ | 247 ± 16 ^b^ |
| ** Cdkn1a* (1387391_at) | 155 ± 34 | 108 ± 34 | 38 ± 17 ^a^ | 31 ± 10 ^a^ |
| ** Cdkn1a* (1388674_at) | 378 ± 44 | 351 ± 33 | 178 ± 18 ^b^ | 165 ± 15 ^b^ |
| *Id2* (1368870_at) | 100 ± 6 | 97 ± 12 | 62 ± 6 ^b^ | 59 ± 4 ^a^ |
| Nucleotide metabolism: |  |  |  |  |
| *Ak3* (1368095_at) | 490 ± 31 | 548 ± 32 | 371 ± 26 ^a^ | 315 ± 25 ^b^ |
| *Nt5e* (1369200_at) | 90 ± 9 | 99 ± 4 | 108 ± 8 | 101 ± 11 |
| *Nt5e* (1384112_at) | 189 ± 9 | 178 ± 14 | 210 ± 15 | 201 ± 5 |
| Apoptosis: |  |  |  |  |
| *# Bnip3* (1387805_at) | 1779 ± 22 | 2238 ± 46 ^b^ | 2148 ± 129 ^a^ | 2803 ± 110 ^b^ |
| ** Bnip3l* (1386978_at) | 542 ± 51 | 797 ± 32 ^b^ | 748 ± 15 ^b^ | 721 ± 19 ^b^ |
| ** Bnip3l* (1367898_at) | 684 ± 29 | 900 ± 40 ^b^ | 842 ± 29 ^b^ | 802 ± 23 ^a^ |
| *# Ddit4* (1368025_at) | 397 ± 47 | 401 ± 37 | 291 ± 5 | 389 ± 35 |
| *Mcl1* (1372520_at) | 899 ± 85 | 636 ± 31 ^b^ | 559 ± 13 ^b^ | 729 ± 39 ^a^ |
| Response to drug: |  |  |  |  |
| *# Abcb1* (1370464_at) | 45 ± 5 | 53 ± 2 | 8 ± 2 ^b^ | 30 ± 10 |
| *# Abcb1* (1370583_s_at) | 165 ± 16 | 194 ± 22 | 43 ± 7 ^b^ | 47 ± 5 ^b^ |
| Circadian clock: |  |  |  |  |
| *Bhlhe40* (1369415_at) | 89 ± 16 | 59 ± 3 ^a^ | 35 ± 4 ^b^ | 126 ± 9 ^a^ |
| *Bhlhe40* (1379483_at) | 322 ± 64 | 207 ± 37 | 117 ± 28 | 393 ± 82 |
| *Bhlhe41* (1368511_at) | 3220 ± 264 | 2614 ± 172 | 2271 ± 257 ^a^ | 2302 ± 102 ^a^ |
| Chemokine activity: |  |  |  |  |
| ** Cxcl12* (1369633_at) | 46 ± 2 | 53 ± 6 | 51 ± 6 | 42 ± 6 |
| ** Cxcr4* (1389244_x_at) | 131 ± 19 | 128 ± 34 | 156 ± 47 | 133 ± 35 |
| ** Cxcr4* (1370097_a_at) | 96 ± 17 | 104 ± 18 | 96 ± 25 | 96 ± 26 |
| ** Cxcr4* (1373661_a_at) | 115 ± 20 | 127 ± 30 | 128 ± 40 | 102 ± 25 |
| Oxidative stress response: |  |  |  |  |
| *Gpx3* (1369926_at) | 303 ± 50 | 385 ± 51 | 429 ± 62 | 365 ± 35 |
| *Hmox1* (1370080_at) | 1252 ± 151 | 765 ± 130 | 264 ± 83 ^b^ | 1058 ± 219 |
| ER chaperones: |  |  |  |  |
| *Grp94* (1388331_at) | 3691 ± 154 | 4116 ± 66 | 4982 ± 109 ^b^ | 5932 ± 192 ^b^ |
| *Hyou1* (1370665_at) | 262 ± 42 | 227 ± 24 | 371 ± 28 | 577 ± 47 ^b^ |
| *Hyou1* (1371442_at) | 755 ± 83 | 705 ± 77 | 1045 ± 75 | 1519 ± 142 ^b^ |
| Others: |  |  |  |  |
| *Cd99* (1371918_at) | 200 ± 31 | 249 ± 20 | 375 ± 29 ^a^ | 312 ± 21 ^b^ |
| *Cited2* (1367602_at) | 631 ± 55 | 481 ± 21 ^a^ | 458 ± 33 ^a^ | 769 ± 41 ^a^ |
| *Cited2* (1367601_at) | 273 ± 25 | 226 ± 15 | 226 ± 23 | 371 ± 29 ^a^ |
| *# Col5a1* (1376099_at) | 79 ± 11 | 94 ± 15 | 114 ± 6 | 122 ± 23 |
| *# Cp* (1368418_a_at) | 45 ± 5 | 51 ± 4 | 37 ± 6 | 35 ± 6 |
| *Ctsd* (1367651_at) | 634 ± 28 | 852 ± 50 ^a^ | 814 ± 47 ^a^ | 696 ± 58 |
| *# Ets1* (1368851_at) | 105 ± 10 | 116 ± 13 | 127 ± 7 | 103 ± 8 |
| *Fech* (1390388_at) | 180 ± 11 | 232 ± 7 | 197 ± 14 | 213 ± 27 |
| *Fn1* (1370234_at) | 282 ± 66 | 320 ± 69 | 519 ± 102 | 772 ± 77 ^b^ |
| *Furin* (1367778_at) | 157 ± 4 | 154 ± 9 | 186 ± 4 ^b^ | 163 ± 2 |
| *Igf2* (1367571_a_at) | 476 ± 31 | 551 ± 33 | 621 ± 53 | 522 ± 23 |
| *# Igfbp1* (1368160_at) | 161 ± 18 | 215 ± 14 | 228 ± 16 | 286 ± 32 ^b^ |
| *Igfbp2* (1367648_at) | 105 ± 16 | 113 ± 21 | 115 ± 29 | 88 ± 17 |
| *Igfbp3* (1367652_at) | 54 ± 10 | 42 ± 10 | 51 ± 18 | 46 ± 16 |
| *Krt18* (1388155_at) | 1739 ± 47 | 1680 ± 96 | 1742 ± 101 | 1866 ± 53 |
| *# Krt19* (1388433_at) | 70 ± 10 | 72 ± 7 | 60 ± 9 | 46 ± 7 |
| *Met* (1369218_at) | 64 ± 5 | 75 ± 15 | 73 ± 11 | 62 ± 5 |
| *Mmp14* (1367860_a_at) | 155 ± 18 | 168 ± 15 | 170 ± 32 | 174 ± 22 |
| *Mmp2* (1370301_at) | 77 ± 11 | 85 ± 8 | 93 ± 14 | 94 ± 18 |
| ** Mxi1* (1368963_at) | 122 ± 10 | 129 ± 15 | 121 ± 9 | 99 ± 12 |
| ** Mxi1* (1372093_at) | 340 ± 20 | 417 ± 13 ^b^ | 324 ± 19 | 256 ± 11 ^b^ |
| *Npm1* (1398756_at) | 246 ± 13 | 327 ± 11 ^b^ | 234 ± 6 | 408 ± 23 ^b^ |
| *Npm1* (1398757_at) | 3148 ± 80 | 3579 ± 119 | 2850 ± 148 | 3247 ± 128 |
| *Npm1* (1399158_a_at) | 2461 ± 222 | 2477 ± 190 | 1426 ± 102 ^b^ | 1811 ± 148 ^a^ |
| *Nr4a1* (1386935_at) | 50 ± 1 | 52 ± 3 | 83 ± 12 | 61 ± 11 |
| *Pdgfa* (1379375_at) | 104 ± 6 | 89 ± 7 | 80 ± 6 | 85 ± 4 |
| *Pdgfa* (1370427_at) | 121 ± 8 | 105 ± 11 | 61 ± 3 ^b^ | 76 ± 5 ^b^ |
| *Ph-4* (1389447_at) | 436 ± 2 | 404 ± 11 ^a^ | 286 ± 8 ^b^ | 283 ± 8 ^b^ |
| *Ppp5c* (1398242_at) | 168 ± 14 | 170 ± 10 | 126 ± 7 ^a^ | 131 ± 6 ^a^ |
| *Tff3* (1387218_at) | 156 ± 9 | 154 ± 12 | 160 ± 13 | 146 ± 12 |
| *Tfrc* (1388750_at) | 778 ± 132 | 661 ± 132 | 492 ± 65 | 693 ± 85 |
| *Tfrc* (1371113_a_at) | 275 ± 33 | 216 ± 25 | 140 ± 5 ^b^ | 198 ± 21 |
| *Tgfa* (1387450_at) | 48 ± 5 | 53 ± 4 | 44 ± 3 | 35 ± 1 |
| *Tgfa* (1381449_s_at) | 192 ± 16 | 175 ± 13 | 159 ± 8 | 113 ± 6 ^b^ |
| *# Tgm2* (1369943_at) | 102 ± 7 | 113 ± 11 | 176 ± 9 ^b^ | 144 ± 8 ^a^ |
| *# Vim* (1367574_at) | 385 ± 75 | 459 ± 100 | 580 ± 142 | 434 ± 100 |
