# Supplementary material for: Glucose-Induced O2 Consumption Activates Hypoxia Inducible Factors 1 and 2 in Rat Insulin-Secreting Pancreatic Beta-Cells
Source: PLoS One. 2012 Jan 3;7(1):e29807. doi: 10.1371/journal.pone.0029807 (PMC3250482; doi:10.1371/journal.pone.0029807)
Supplement: Table S2 — Sequences of oligonucleotide primers and reaction conditions for real-time PCR amplification of rat cDNA, and characteristics of PCR products. Tm: Amplicon melting temperature; a: Islet sample cDNA quantity per tube (ng total RNA equivalent). (DOC) [file pone.0029807.s006.doc]

| **Gene** | **Sense primer (5’-3’)** | **Antisense primer (5’-3’)** | **Input ^a^** | **Annealing** | **Extension** | **Amplicon size** | **Tm** |
| --- | --- | --- | --- | --- | --- | --- | --- |
|  |  |  |  | **(°C-sec)** | **(°C-sec)** | **(bp)** | **(°C)** |
| *Tbp* | ACC.CTT.CAC.CAA.TGA.CTC.CTA.TG | ACT.TCG.TGC.CAG.AAA.TGC.TGA | 2 | variable | variable | 157 | 84 |
| *Hif1α* | tgg.tgc.tga.ttt.gtg.aac.cca.ttc.c | cag.ggc.atc.ggg.ctc.ttt.ctt.aag | 2-4 | 60-90 | none | 533 | 83 |
| *Hif2α* | ggt.gac.cca.aga.cgg.tga.tat.gat.c | aac.cag.agc.cag.ttt.tga.gtg.tca.g | 2-4 | 60-90 | none | 161 | 84.5 |
| *Arnt* | acc.att.gtc.cag.ccg.tca.tc | cga.gtg.cta.gag.gtc.caa.gtt | 4 | 60-45 | 77-15 | 104 | 84.5 |
| *Adm* | gtt.tcc.atc.gcc.ctg.atg.tta.ttg | gct.gct.gga.cgc.ttg.tag.ttc | 2-10 | 60-45 | 80-15 | 144 | 87.5 |
| *Aldoa* | gtc.cct.tcc.ccc.aag.tta.tc | cgt.cca.gcc.ctt.gag.tag.tg | 2 | 60-45 | none | 114 | 85 |
| *Tpi1* | cat.tgg.gga.gaa.gtt.aga.cga.aag | ccg.gag.ctt.ctc.gtg.tac.ttc | 2-4 | 60-45 | 82-15 | 190 | 86 |
| *Gapdh* | GTC.GGT.GTC.AAC.GGA.TTT.GG | CGT.GGG.TAG.AAT.CAT.ACT.GGA.ACA.T | 2 | 62-45 | 82-15 | 145 | 87 |
| *Eno1* | cgg.tcc.cag.ctt.tca.atg.tg | tgg.cgt.ctt.tcc.cgt.act.tc | 2 | 60-45 | none | 177 | 86.5 |
| *Pkm2* | ctg.ctg.ttt.gaa.gag.ctt.gcg | act.ccg.tca.gaa.cta.tca.aag.ctg | 2 | 60-45 | 80-15 | 124 | 88 |
| *Ldha* | TGC.TGG.AGC.CAC.TGT.CG | CTG.GGT.TTG.AGA.CGA.TGA.GC | 4 | 62-90 | 83-15 | 518 | 87.5 |
| *Mct4* | cgg.tct.ttg.tgg.tga.gct.atg.c | gca.cct.tct.tga.gcc.ctg.tta.tg | 10 | 60-45 | none | 135 | 86.5 |
| *Pdk1* | gtt.ccg.tcc.cat.ctc.tat.cac.a | gaa.ctt.gaa.tcg.ggg.gat.aaa.cg | 2-4 | 62-45 | 76-15 | 106 | 81 |
| *Car12* | cGC.TAC.TGT.CGT.CCT.CCT.ATT.G | CAC.ATG.ACG.GGT.ACT.TCT.TGG.A | 10 | 62-45 | 82-15 | 131 | 85.5 |
