# Supplementary material for: Glucose-Induced O2 Consumption Activates Hypoxia Inducible Factors 1 and 2 in Rat Insulin-Secreting Pancreatic Beta-Cells
Source: PLoS One. 2012 Jan 3;7(1):e29807. doi: 10.1371/journal.pone.0029807 (PMC3250482; doi:10.1371/journal.pone.0029807)
Supplement: Table S3 — Sequences of oligonucleotide primers for real-time PCR amplification of mouse cDNA. (DOC) [file pone.0029807.s007.doc]

| **Gene** | **Sense primer (5’-3’)** | **Antisense primer (5’-3’)** | **Product (bp)** |
| --- | --- | --- | --- |
| *Hif1α* | CAT.CTC.TCT.GGA.TTT.TGG.CAG.C | GAA.GTG.GCT.TTG.GAG.TTT.CCG | 161 |
| *Hif2α* | CGC.TGC.TCT.CTC.TCT.CTT.TTG.G | GCT.CAA.TCA.GGT.GGA.AGT.TTG.C | 152 |
| *Adm* | GCA.ATG.CTT.GTT.GTC.CAG.CC | ACA.CAC.ACA.CAC.ACA.CAC.GGA.AC | 219 |
| *Aldoa* | TCC.ATT.GGC.ACC.GAG.AAC.AC | TTG.ATA.ACT.TGG.GGG.AAG.GGA.C | 161 |
| *Tpi1* | CCT.TCC.ATT.GGT.TTG.GGC.TG | AAT.ACA.GGG.GCT.TTG.GCA.CC | 222 |
| *Gapdh* | CAA.AAT.GGT.GAA.GGT.CGG.TGT.G | TGA.TGT.TAG.TGG.GGT.CTC.GCT.C | 254 |
| *Eno1* | GAT.GGA.CGG.CAC.AGA.GAA.TAA.ATC | AGG.CAG.GAT.GAC.TTC.AGG.GTT.G | 151 |
| *Hyou1* | TAC.TCC.CGT.TCC.TTG.GCT.GAA.G | GGC.TGT.GGC.AGT.GTT.GTC.ATT.G | 165 |
